# Reinterpreting patterns of variation in human thyroid function: An evolutionary ecology perspective

**DOI:** 10.1093/emph/eoaa043

**Published:** 2020-11-10

**Authors:** Sarai Keestra, Vedrana Högqvist Tabor, Alexandra Alvergne

**Affiliations:** 1 School of Anthropology & Museum Ethnography, University of Oxford, Oxford, UK; 2 Amsterdam UMC, University of Amsterdam, Amsterdam, The Netherlands; 3 BOOST Thyroid by VLM Health, Berlin, Germany; 4 ISEM, Université de Montpellier, CNRS, IRD, EPHE, Montpellier, France

**Keywords:** evolutionary medicine, thyroid hormones, hypothyroidism, hyperthyroidism, autoimmune thyroid diseases, evolutionary ecology

## Abstract

Two hundred million people worldwide experience some form of thyroid disorder, with women being especially at risk. However, why human thyroid function varies between populations, individuals, and across the lifespan has attracted little research to date. This limits our ability to evaluate the conditions under which patterns of variation in thyroid function are best understood as ‘normal’ or ‘pathological’. In this review, we aim to spark interest in research aimed at understanding the causes of variation in thyroid phenotypes. We start by assessing the biomedical literature on thyroid imbalance to discuss the validity of existing reference intervals for diagnosis and treatment across individuals and populations. We then propose an evolutionary ecological framework for understanding the phylogenetic, genetic, ecological, developmental, and physiological causes of normal variation in thyroid function. We build on this approach to suggest testable predictions for how environmental challenges interact with individual circumstances to influence the onset of thyroid disorders. We propose that dietary changes, ecological disruptions of co-evolutionary processes during pregnancy and with pathogens, emerging infections, and exacerbated stress responses can contribute to explaining the onset of thyroid diseases. For patients to receive the best personalized care, research into the causes of thyroid variation at multiple levels is needed.

## Lay Summary

Thyroid hormone reference intervals—used to determine normal thyroid function —currently don’t take into account many significant factors that can cause variation in thyroid hormone levels. These factors include age, sex, ethnicity, season, time of day, iodine content in the diet, socioeconomic status, stress levels, body composition, immune status, menstrual cycle phase, and overall health status. This paper shows how early life experiences as well as short term stressors may affect variation in thyroid function. These are energetic challenges to which the thyroid physiology can respond to. Our investigation shows that much variation in thyroid function is natural. It may result from a complex interplay of evolutionary, genetic, developmental, and physiological factors in response to energetic challenges in the environment, beyond what is currently considered in biomedicine. A new research agenda for thyroid health should explore the way that diversity in thyroid function has evolved as a response to different contexts people live in—like focusing on how people’s metabolisms adapt to the energetic requirements of their environments.

## INTRODUCTION

Thyroid dysfunction affects 200 million people worldwide [1]. Although most research is conducted in Westernized, relatively wealthy societies, thyroid diseases are surprisingly common in all populations studied [2]. Thyroid function abnormalities have significant ramifications for body temperature regulation [3], metabolism [4], cardiac function and blood pressure [5], fertility [6], foetal neurological development [7], intellectual performance of school-aged children [8], mental health [9], and overall quality of life [10]. Even at subclinical levels, thyroid dysfunction is associated with stroke risk [11], cardiac dysfunction [5], and neuro-psychiatric disorders, including anxiety and depression [12, 13]. Understanding the causes underpinning thyroid dysfunction is therefore critical to improve global health.

This paper provides a holistic framework for rethinking the causes of variation in thyroid function at multiple levels (i.e. between species, populations, individuals, and across the lifespan). We aim to spark interest in reconsidering what counts as ‘normal’ and ‘pathological’ in thyroid phenotypes. While health is commonly defined as “a state of complete physical, social and mental wellbeing [14], from an evolutionary perspective, health is a means to the end of reproduction. In this latter framework, health is better conceptualized as the ability to adapt to changing environments [15]. As a result, patterns of variation in thyroid function are expected to be constrained by phylogeny, genetic polymorphism, variation in early environments, and current ecological stresses, which renders the task of differentiating between ‘normal’ and ‘pathological’ states difficult. To shed new light on patterns of variation, we first outline the biomedical literature on imbalances in the hypothalamic–pituitary–thyroidal (HPT) axis (Box 1), arguing that the use of reference intervals based on large population samples obscures natural intra-individual variation in thyroid function. Second, we review the ultimate (i.e. evolutionary) causes of variation in thyroid function, including phylogenetic and genetic influences, to show how the thyroid system accumulated multiple roles that are both common to eukaryotes and specific to taxa, species and human populations. Third, we propose a life-history framework for understanding variation in thyroid function in response to environmental stressors experienced at various life stages. Fourth, we apply this multi-level framework to pathological patterns of thyroid function variation, focusing on four case studies (i) mismatches in diet composition and lifestyle, (ii), co-evolutionary processes around pregnancy, (iii) microbial exposure and emerging infections and (iv) exacerbated stress responses. We contend that by understanding the role of thyroid function in regulating the energetic trade-offs between the functions of reproduction, growth, and somatic maintenance, an evolutionary medicine approach can contribute to clinical medicine by reinterpreting natural variation in thyroid function within an ecological context. We conclude that field studies in different ecologies are needed to elucidate natural variation of human thyroid function, which is crucial for devising appropriate reference intervals.

Box 1. **The hypothalamic–pituitary–thyroid (HPT) axis and the diagnosis of thyroid dysfunction**

Thyroid hormone production is under the regulation of the **hypothalamic–pituitary–thyroid (HPT) axis**, which starts with hypothalamic neurons secreting **thyrotropin-releasing hormone (TRH)**, stimulating the release of **thyroid-stimulating hormone (TSH)** from the pituitary. TSH binds to receptors on the thyroid, which produces two closely related thyroid hormones**; thyroxine (T4)**, and **triiodothyronine T3**. These thyroid hormones are synthesized through the iodination of the amino acid tyrosine by the enzyme **thyroid peroxidase (TPO)**, and can consequently temporarily be stored as an intermediate **thyroglobulin** complex. The majority of the thyroid gland’s output consists of T4, which is the less metabolically active variant of T3, but binds to thyroid hormone binding proteins with higher affinity, and therefore has a longer half-life in the circulation. Unbound, free thyroid hormone is taken up by cells, where they have a wide range of genomic and non-genomic effects, including the enhancement of oxygen use in the mitochondria, increasing energy availability for muscular work, and thermoregulation. Through differential expression of **deiodinase enzymes**, which convert the pro-hormone T4 to its active T3 form, tissues can adjust the concentration ratio of free thyroid hormones to local metabolic needs.

Thyroid dysfunction is commonly diagnosed by analysing serum **thyroid-stimulating hormone (TSH)**, and often other thyroid biomarkers are only tested if TSH is considered abnormal. **TSH** tests are then complemented by measurements of thyroid **autoantibodies**, free **thyroxine (T4)**, and its more metabolically active form free **triiodothyronine (T3)**. These biomarkers are compared with reference intervals for large populations without thyroid disease, using a 95% interval for **T4** and **T3** and the 2.5th and 97.5th percentiles to determine the normal **TSH** reference range [31]. **T4** and **T3** are produced in response to TSH under negative feedback from the **hypothalamic–pituitary–thyroidal (HPT)** axis [22]. A hypothyroid state is diagnosed when **TSH** levels are elevated and free T4 is decreased as compared to the reference interval [5], whereas hyperthyroidism is diagnosed if **TSH** levels are decreased and free T4 levels are elevated [4, 39]. The diagnosis and treatment of thyroid dysfunction is often considered simple and done in a primary care setting [35].

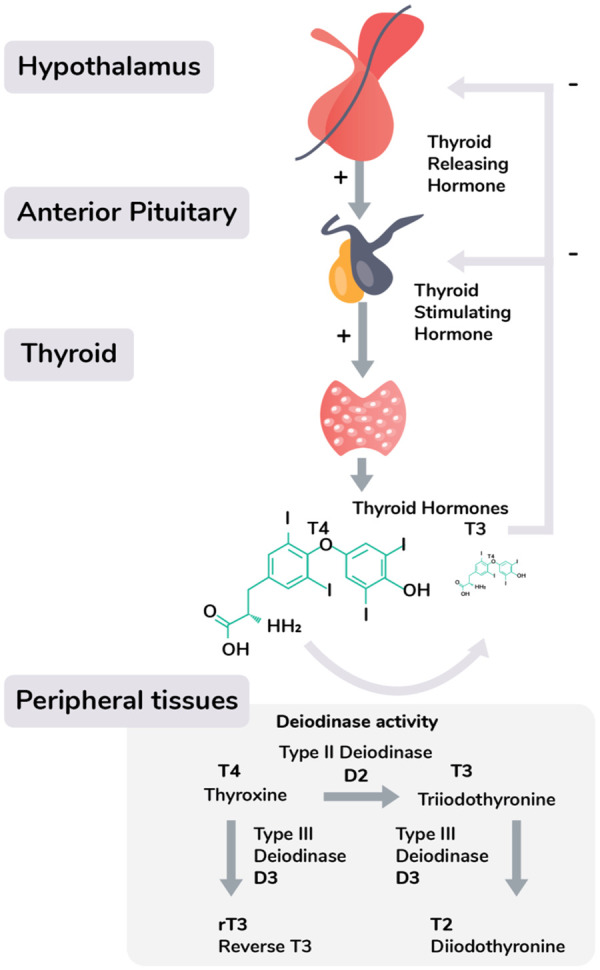

### 1. Biomedical approaches to thyroid function

#### Thyroid diseases: causes and treatment

From a biomedical perspective, the main causes of thyroid dysfunction are thyroid cancer, **autoimmune thyroid diseases (AITD)**, and deficiencies in iodine and certain other micronutrients. Thyroid cancers are the most prevalent endocrine tumour [16], and despite being classified as non-reproductive cancers, thyroid neoplasms occur more often in women than men [17], and are the most commonly diagnosed cancer during pregnancy after breast cancer [18]. affects women nine times more than males [19], and autoimmunity leading to underactive thyroid function (**hypothyroidism**) is the most common of all autoimmune and endocrine disorders [20]. **Hashimoto’s thyroiditis**, a condition characterized by chronic inflammation and the presence of **thyroperoxidase** (**TPO**) and **thyroglobulin** (**TG**) **autoantibodies**, causes the destruction of the thyroid gland and **hypothyroidism** [21]. In **Graves’ disease**, a mix of stimulating and blocking **autoantibodies** against the TSH-receptor overstimulates thyroid growth and hormone production, most often leading to **hyperthyroidism**, and in rare cases, hypothyroid or euthyroid Graves’ disease [4, 22, 23]. Worldwide, more than 1 in 20 individuals has a subclinical underactive thyroid [24], and two billion people are at risk of suffering from the health consequences of deficiencies in iodine [25], a micronutrient needed for the synthesis of thyroid hormones. Iodine deficiency disorders commonly manifest themselves through **hypothyroidism** and endemic **goitre**, but can also cause wide-ranging deficits in cognition, growth, and fertility, due to thyroid hormones’ vital importance for normal development, especially of the brain [26]. In severe iodine-deficient regions, endemic **cretinism** persists until the present day, manifesting itself as a neurological disorder with brain damage, mental retardation, and deaf mutism due **maternal hypothyroxinemia**, often caused by iodine deficiency, during gestation. **Neurological cretinism** occurs alongside **myxedematous cretinism**, which results from severe **hypothyroidism** as a consequence of **athyreosis** or an underdevelopment of the thyroid at birth, causing dwarfism and impaired neurodevelopment if not diagnosed and corrected early using thyroid hormone replacement therapy [26–29].

Treatment for thyroid dysfunction does not always produce the expected outcomes. For hyperthyroid individuals, surgery, antithyroid drugs, or radioiodine treatment are used to bring thyroid hormone production back into the strictly defined reference range [5, 30, 31]. However, often, too much of the thyroid is inactivated, which is why 80% of hyperthyroid patients become hypothyroid after treatment [32]. Furthermore, many individuals receiving lifelong **T4** replacement therapy (**levothyroxine**) to correct underactive thyroid function continue being hypothyroid or become hyperthyroid due to overmedication [24]. A third of patients taking levothyroxine have abnormally high free **thyroxine** (**T4**) compared to free **triiodothyronine** (**T3**) ratios and up to 10% of patients experience depression, anxiety, or other manifestations of impaired psychological well-being despite normal TSH levels [33]. Many patients therefore continue to experience a decreased quality of life even after thyroid function tests are in the right range according to reference values [34]. This raises the question of how reference intervals, the decision support tool for the interpretation of biomarkers, are developed.

#### Reference intervals: what is the ‘normal’ range?

**Figure 1.**
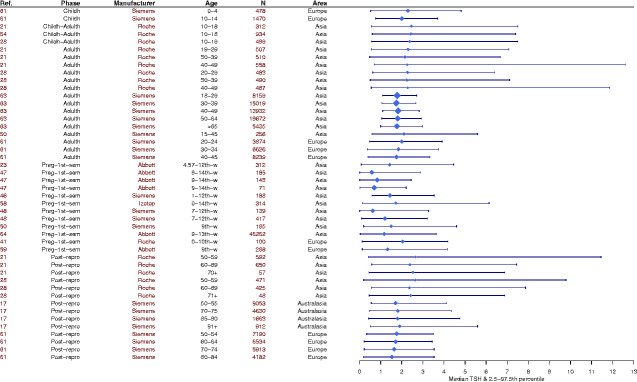
Global variation in female TSH reference intervals across reproductive life stages. Because of the way reference ranges are constructed, the 2.5–97.5th percentiles and medians of TSH measurements in different populations across the world will vary depending on the geographical location, ethnicity of the participants, age and reproductive life stage of the participants, assay manufacturer, statistical method used, and the laboratory where the test has been conducted. We chose to look at female reference ranges only because thyroid disorders are more prevalent in women and most studies report a significant difference between female and male TSH reference intervals. Where possible we display the disease-free population to reflect the diversity of normal, ‘healthy’ TSH reference intervals used for women around the world. In this figure, we only display a subset of the TSH reference intervals that were published between 2017 and early 2020. For further information on the methods used in the systematic search to create this figure and the references for the intervals displayed, please see Supplementary Table S1.

The idea of a ‘normal range’ for thyroid biomarkers is debatable on both methodological and biological grounds. Despite recent attempts to harmonize TSH reference intervals across laboratories and countries [35], there is still significant variation in assay sensitivity across manufacturers [36], differences in protocols for thyroid biomarkers analysis between laboratories [37], disagreement over the appropriate statistical methods to establish reference intervals, and disparities in manufacturers’ proposed reference intervals [31]. Furthermore, the half-lives of **TSH** and **T4** in the blood differ (one hour vs one week in the circulation [38]), thus assessing the thyroid status of individuals currently experiencing changes in energy balance relying on these biomarkers alone is particularly challenging [39]. Finally, reference intervals vary significantly depending on the population and assay manufacturer used to construct them (Fig. 1, Supplementary Table S1) [35].

From a biological perspective, reference intervals should be adjusted to account for the significant inter- and intra-individual variation in thyroid function. Although it is known that thyroid function varies widely with age [40, 41], sex [42], ethnicity [43], season [44], time of the day [45, 46], iodine sufficiency of the region [2, 47], socioeconomic status [48], stress levels [49], body mass index (BMI) [50], white blood cell count [51], menstrual cycle phase [52], overall health status [53], and is released in a pulsatile fashion [54], these factors are not typically taken into account by clinicians in the diagnosis of thyroid disorders, nor is the treatment adjusted accordingly [55]. Yet a single blood test needs to be adjusted by ±25% for thyroid hormones and ±50% for **TSH**, as repeated testing of healthy subjects reveals significant intra-individual variation around a unique, personal thyroid function [56]. Furthermore, while thyroid function tests might sometimes fall outside of the reference interval, in the absence of symptoms, they might not always indicate that medication is necessary [31]. Conversely, small variations in thyroid function can be experienced as significant to the individual, but reference ranges that are constructed relying on population level variation obscure this individual variation [56–58].

By not taking into account the possibility that variation in thyroid hormones could be the manifestation of an evolved response to individual environmental contexts, biomedicine runs the risk of treating the symptoms of the problem instead of addressing the underlying causes of thyroid ill-health. In the remainder of the paper, we draw on an evolutionary ecological framework to shed new light on the causes of variation in thyroid function. In such a framework, variability is the norm ‘rather than an aberration’ [59] and various interconnected levels of causality must be considered for understanding both normal and pathological patterns of variation, including evolutionary, ecological, developmental, and physiological factors [60].

### 2. The evolutionary history of thyroid function

**Figure 2.**
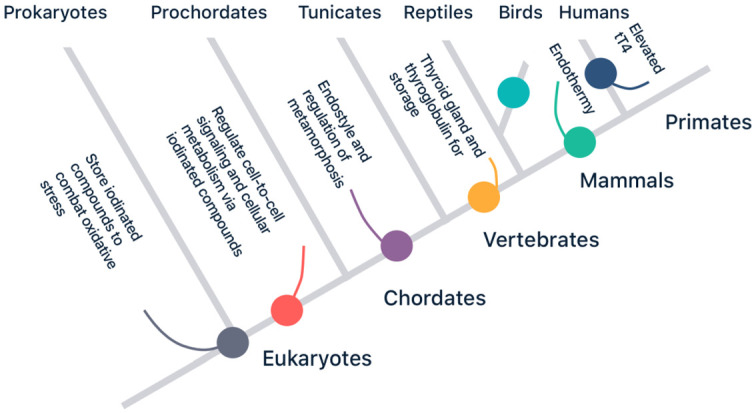
A simplified phylogeny of thyroid function. The ability to use of iodine, iodides and iodinated compounds is thought to date back as far as the prokaryote ancestor of all eukaryote life. Later, these molecules might have played an important role in the evolution of multi-cellular life, cell-to-cell signalling, and the regulation of cellular metabolism. The endostyle, which is the precursor of the vertebrate thyroid gland, developed in chordates circa 500 million years ago. The evolution of the enzymatic machinery of the endostyle may have been vital for the evolution of terrestrial life. All vertebrates share a thyroid gland and thyroid hormones with a similar chemical structure, however in both birds and mammals thyroid function was further upregulated throughout evolution leading to endothermy, the ability to regulate body temperature. Finally, in humans further selection pressure on the thyroid led to elevated total T4 concentrations compared to our Chimpanzee cousins.

Normal patterns of variation in thyroid function are constrained by the evolutionary history of the thyroid system. Whilst the initial use of thyroid hormone precursors most likely evolved to counteract oxidative stress in unicellular organisms, the capacity of thyroid hormones to regulate metabolism has since been co-opted multiple times (Fig. 2), e.g. to coordinate development in chordates and facilitate the evolution of **endothermy** in birds and mammals. In humans, there is additionally some evidence for genetic polymorphisms evolved in response to cold and iodine-deficient ecologies (Box 2).

Box 2. **Human genetic polymorphism in thyroid function**

Throughout human evolution, various evolutionary processes, including natural selection, likely shaped diversity in human thyroid physiology across populations living in different geographies in the face of different ecological pressures [164].

***Adaptation to iodine-deficient environments***. The Ituri forest in the Congo basin is an iodine-deficient region with high prevalence of goitre (42.9%) in the village-dwelling Bantu population, yet Efe pygmees, who have inhabited this region for ten thousands of years, suffer from much lower goitre rates (9.4%) than expected[165]. Genetic studies have found two genes involved in the thyroid hormone pathway showing strong signatures for positive selection in Mbuti and Baiaka pygmies, who are closely related to the Efe and live in the same forest [166]. This suggests that these populations may have adapted to an iodine-deficient diet through genetic adaptation [164]. Whether other populations have evolved other genetic or cultural adaptations in response to similar dietary challenges, such as in the case of lactose intolerance where various populations evolved different adaptations, would be an interesting avenue to explore in future research.

***Adaptation to cold environments*** Basal metabolism rates are closely associated with T4 levels in indigenous Evenki herders and Russians living in the arctic temperatures of Siberia [79]. Yet, Evenki women display higher free T4 levels than Russian women living in the same area, which could be an evolutionary advantageous adaptation to the cold temperatures of the Siberian winters by elevating metabolism rates [79, 167]. Investigations of the mitochondrial DNA of Siberian populations have furthermore shown a strong selection on genetic variants that contribute to enhanced metabolic heat production, which also suggests that their mitochondria might react more strongly to thyroid hormones’ metabolic influences [79, 168].

While various single nucleotide polymorphisms exists in genes coding for the deiodinase enzymes, as well as the **TSH** and **T3** receptors, corresponding to differences in **TSH** and **T3** levels [164, 169], there is currently a dearth of research on the historical and ecological causes for genetic polymorphism in thyroid function in humans. Further genetic studies in various localities are needed to elucidate how evolutionary processes (natural selection, drift, migration, mating) acting on genetic variants have contributed to genetic polymorphism in thyroid function.

Thyroid hormones have acquired a wide range of functions during their evolutionary history through **exaptation** (Fig. 2). The cellular use of iodine and its hydrated derivatives, iodides, predates eukaryotic life itself and may have been present in prokaryote oxygen-producing cyanobacteria living three billion years ago [61, 62]. Iodinated compounds, abundant in the primordial sea, probably served as antioxidants against the damaging effects of radical oxygen species, which in the form of oxidative stress can interfere with normal cellular signalling, growth, differentiation, and metabolism [63]. Because these cyanobacteria were later incorporated in eukaryotic cells as mitochondria, iodinated tyrosines, the evolutionary and biochemical precursors of thyroid hormones, have important regulatory roles in cellular metabolism of all eukaryotes [62]. Algae, one of the most ancient forms of multicellular eukaryote life, accumulate large quantities of iodine from sea water, and various other invertebrates store iodinated tyrosines [64]. As iodinated tyrosines easily pass through cell membranes, they were potentially involved in the earliest forms of cell-to-cell signalling and the evolution of multicellular life. Already in the common ancestor of invertebrates and vertebrates the presence of iodotyrosines may have served as an energy-related **plasticity** cue [62, 67]. Iodine additionally plays a role in the most primitive features of the immune system, by increasing inflammation and enhancing phagocytosis [65]. Some non-genomic functions of iodinated compounds therefore precede thyroid hormones and are shared with invertebrates and vertebrates alike.

Thyroid hormones later developed novel, genomic functions in the coordination of development [64, 68]. Although non-vertebrates use exogenous thyroid hormones derived from food (e.g. ingested algae) as a developmental cue of energetic conditions to initiate metamorphic transformation, in vertebrates these thyroid hormones are produced endogenously for the first time [62, 67]. Experiments in the early 20th century established the important role of the thyroid in the timing of metamorphosis of tadpoles into frogs [69]. Further research has shown that thyroid hormone signalling coordinates the timing of tissue differentiation programmes across different chordate species, including human neurodevelopment during gestation [7]. Crockford [54, 62] and others have argued that due to their central regulatory role and pleiotropic actions in growth, development, reproduction, and stress, alterations in thyroid phenotypes may subsequently underlie rapid speciation and the gradual adaptation of populations to new habitats.

The evolution of the thyroid gland may have enabled the evolution of terrestrial life by storing a pool of releasable thyroid hormone precursors in order to compensate for the unpredictable access to iodine on land [22, 62, 70, 71], where it may only be found in certain foods such as dark green leafy vegetables, eggs, and seeds (see also Section 4). Originally, thyroid-hormone producing cells, organized in follicles, where scattered throughout the body until the emergence of the precursor of the thyroid gland, the **endostyle**, which developed from the primitive gut of chordates and evolved the enzymatic machinery found in all vertebrate thyroids today [70, 72, 73]. The evolutionary origin of the gland from the foregut can be witnessed in the human embryology of the thyroid, as the thyroid develops from the endodermal cells at the base of the tongue and then descends downwards to its position in the neck [74].

In contrast to many other hormonal systems, all vertebrate species essentially share the same chemical structure for thyroid hormones and their active derivatives [66]. The evolution of the follicular thyroid in vertebrates might have been critical to allow for life to move from saltwater to iodine-depleted freshwater to land. Such transition was facilitated by both the biochemical evolution of cell surface iodine transporters, driving iodine against its concentration gradient, and the appearance of the **TPO** enzymatic machinery, which allowed the iodine to be stored as **thyroglobulin** complexes, an intermediary product of thyroid hormones [61, 62, 72]. By binding most of these hydrophobic thyroid hormones to hydrophilic carrier proteins such as thyroid-binding globulin, albumin, and transthyretin, thyroid hormones can be easily transported in the circulation, prolonging their half-life and metabolic turnover . Yet, even small polymorphisms in genes related to thyroid hormone production and metabolism can lead to significant interspecies variation in thyroid rhythm phenotypes [54]: humans have higher total **T4** and **T3** levels and increased free **T4** levels than other great ape genera [75], which may be due to lower transthyretin levels in humans [76].

Mammals and birds subsequently developed **endothermy** through **convergent evolution** enhancing thyroid-driven metabolism, allowing them to adjust to colder terrestrial environments [54, 72, 77]. Intensified selection on thyroid function and resting metabolic rate may have continued throughout human evolution for example due to the sustained exertion involved in early hunter-gathering activities and the challenges posed by colder climates during human expansions out of Africa [72, 78, 79]. It has even been speculated that in Neanderthals, selection towards a distinct cold-tolerant thyroid hormone phenotype, and its downstream effects on developmental programmes, resulted in significant differences in Neanderthal post-natal growth rates and morphology compared to *Homo sapiens* despite their closeness in genetic ancestry [54].

Climatic changes altered the ecological niche and thereby the dietary intake of iodine and exogenous thyroid hormones ingestion throughout human evolutionary history, which may have played an important role in speciation events in the human lineage [54]. Given the importance of thyroid hormones for cognitive development and function [7], some authors have proposed that enhanced access to iodine-rich foods due to increased meat consumption or a shore-based diet may have freed human brain development from the nutritional constraints on thyroid function experienced by other primates [28, 80–82]. For example, the shift from scavenging towards hunting in an open savannah-type environment that accompanied the emergence of *Homo erectus* would have increased thyroid gland consumption by hominins and thereby exogenous thyroid exposure, potentially causing heterochronic changes in both body proportions and brain size through altering the timing of developmental programmes [54]. Due to the importance of thyroid function in brain development, and the ability of thyroid hormones to enhance the synthesis of the precursor of the neurotransmitter dopamine, it has even been hypothesized that increasing thyroid hormone levels throughout our evolutionary history has contributed to the evolution of intelligence and increased cognitive capacity in modern day humans compared to our ancestors [82].

The evolutionary literature therefore shows that since the first use of iodides in cyanobacteria, the thyroid system adopted a wide range of functions, from the regulation of cellular metabolism and body temperature to the coordination of tissue differentiation during development. Acknowledging that the thyroid system serves multiple functions evolved in response to various species-specific environmental challenges can help draw a holistic framework in which evolutionary and ecological factors are integrated together to explain patterns of variation.

### 3. The ecology of thyroid function

#### Thyroid function, **plasticity**, and life-history theory

Natural diversity in thyroid hormone levels may result from phenotypic **plasticity**, i.e. the evolved capacity of an organism to adjust its physiology, development, and behaviour to variable environments in a way that maximizes its overall fitness [83]. To understand how organisms ‘adjust’ their phenotype to their environments, evolutionary biologists use **life-history theory** [84, 85]. This framework posits that when resources are limited, organisms face **trade-offs** between the fitness functions of growth, reproduction, and somatic maintenance (Box 3). Natural selection is expected to favour organisms that make optimal allocation ‘decisions’ in growth, reproduction, and immunity, given each life stage (e.g. infancy, childhood, adolescence, adulthood) and ecological conditions (i.e. mortality risk, resource availability).

Box 3. **Integrating thyroid function into a life-history framework**

Thyroid hormones are involved in the regulation of the key fitness functions of growth, reproduction, and immunity. Firstly, thyroid hormones stimulate growth by affecting the expression of genes involved in cellular growth and differentiation, and by stimulating DNA synthesis in osteoblasts and other cells [170]. Secondly, the thyroid axis interacts with reproductive function by influencing the metabolism of oestrogens and androgens and the unbound levels of these sex hormones [171]. In a study of 86 **euthyroid** women in Michigan, who were not lactating or taking hormonal medications, greater total **T4** was associated with higher levels of urinary metabolites of progesterone and oestrogen, whereas at the same time elevated free T4 was associated with a shorter follicular phase and cycle length [172]. Thirdly, thyroid hormones play an important role in somatic maintenance by enhancing oxygen use in the mitochondria, thereby increasing cellular energy availability for muscular work or thermoregulation [4] and stimulating the immune system [173]. As a consequence, individuals with an underactive thyroid often display immune deficits and are left more vulnerable to infection. By contrast, elevated thyroid hormone levels are associated with improved pathogen clearance due to the importance of thyroid hormones for the maturation and differentiation of the innate and adaptive immune cells and for the immune processes of phagocytosis, cytokine production and release [173].

The hypothalamus is the starting point of the four endocrine axes that neatly correspond to the branches of life-history theory of growth, reproduction and somatic maintenance. These are the hypothalamic–pituitary–somatotropic, hypothalamic–pituitary–gonadal, and hypothalamic–pituitary–adrenal axis, respectively, and we are adding the HPT axis as an important addition to this framework proposed by Wang et al. (2019) [174]. The final outputs of these axes are hormones: the HPS-axis governs the pituitary secretion of growth hormone and the hepatic secretion of IGF-1 involved in protein metabolism and growth; the HPG-axis coordinates the release of reproductive hormones from the gonads; and the HPA-axis regulates the adrenal secretion of glucocorticoids in response to environmental stress, adjusting the partitioning of resources between competing demands. By modulating optimal energy investment in peripheral tissues, these hormones are the physiological mediators by which organisms adjust maturation trajectories and modify cellular functions across different tissues and organ systems in response to environmental cues. Through affecting neuroendocrinology of the hypothalamus, early childhood conditions could potentially create new set points for the coordination of the different axes, which might play a role in mediating life-history trade-offs and the timing of key transitions such as maturation.

Considering the role of thyroid hormones in these different physiological axes from a life-history perspective, diversity in thyroid hormone levels can be expected to correlate with energetic investments in growth, reproduction and somatic maintenance over the lifespan. Although other hormones such as sex steroids are also involved in regulating energetic trade-offs, thyroid hormone modulation may play a unique role as it is not necessarily limited to specific life stages, but may act over longer time scales. We therefore argue that the HPT axis should be considered as alongside other key physiological axes that mediate life-history trade-offs and energetic investments over the lifespan [174], especially because of thyroid hormones’ important role in determining basal metabolic rate and thereby long-term energetic expenditure. Lead to various life-history trajectories with regards to growth, reproductive development and immune function. At the physiological level, such allocation ‘decisions’ are regulated through the action of hormones which modify cellular functions across different organs and tissues in response to endogenous as well as exogenous environmental stressors [86]. Given that thyroid hormones play a critical role in modulating energy expenditure and the timing of life-history transitions (e.g. metamorphosis), using a life-history framework for understanding how environmental diversity mediates variation in thyroid function appears relevant.

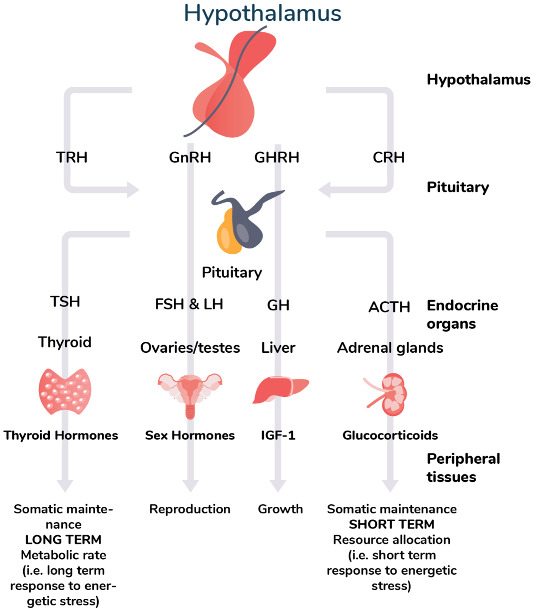

[86].

#### Developmental plasticity: the importance of early life environment

Developmental **plasticity** is a form of phenotypic **plasticity**, which indicates the ability of an organism to adjust its phenotype to the conditions encountered early in life. In this way, variation in thyroid function might correlate with conditions encountered early in life, where environments characterized by high mortality risk promote an accelerated development and metabolism through the upregulation of the thyroid, while early energetic deprivation promote the downregulation of thyroid hormones to slow down maturation and preserve energy for later reproduction (Box 4).

Box 4. **Thyroid function, reproductive development, and the fertility–longevity trade-off**

Research from human reproductive ecology has shown that under conditions of chronic energetic stress early in life, delaying maturation and downregulating ovarian function is sometimes adaptive by yielding the maximum lifetime reproductive output possible in a resource-limited ecology [175]. Given the influence of thyroid function on the reproductive axis, we hypothesize that in energetically challenging circumstances, lowered thyroid function plays a role in determining the pace of reproductive development and fertility. Lowered thyroid function may indeed contribute to delayed pubertal development [21], and although subclinical underactive thyroid function only mildly affects the menstrual cycle [176], for hypothyroid individuals menstruation is often less frequent [177]. Furthermore, women with severe hypothyroidism often fail to ovulate [177] and an underactive thyroid is a common cause of spontaneous abortion during the fertile years [178], although after conception, live birth rate is not always affected [179]. This suggests that an underactive thyroid might lead to an increased interbirth interval, whereas in hyperthyroid women, menstrual cycles are shortened and occur more frequently, but remain ovulatory [180]. It is not clear how far these plastic changes in reproduction can be attributed to constraints in early life or the current environment. Studies in natural fertility populations with various modes of subsistence are necessary to further investigate possible associations between energetic conditions, thyroid function and reproduction.

Thyroid function might also play a key role in mediating the trade-off between longevity (i.e. stress resistance) and reproduction, although this has remained largely unexplored in humans. Studies in Wistar rats suggest that an underactive thyroid might contribute to an increased lifespan by reducing the overall metabolic rate, oxidative stress, and cell senescence whereas artificially inducing hyperthyroidism using **T4** supplementation shortened life duration, most likely due to accelerated ageing [181, 182]. Although much less is known about the effect of hyperthyroidism on lifespan in humans, some studies have noted that, even at subclinical levels, hyperthyroidism is associated with a higher risk of cardiovascular diseases [11, 183], in contrast to subclinical hypothyroidism [5]. Studies showed that centenarians are much more likely to have slightly elevated TSH, as do their closest family members and offspring [181, 184, 185], which suggests that longevity and thyroid function are to some degree inherited [164]. How far these effects might also be due to developmental influences, however, remains unclear.

##### Life-expectancy at birth

When environments are characterized by a high extrinsic risk of mortality (i.e. mortality due to external stressors rather than the phenotype), organisms are expected to display accelerated reproductive development [84, 85], with faster transitions between life stages and the prioritization of reproductive function at the expense of other fitness functions (growth, somatic maintenance). In this line, children growing up in the U.K. and the U.S. in socioeconomically deprived circumstances associated with a shorter life-expectancy undergo reproductive maturation earlier, have their first child at a younger age, and experience poorer quality of health [87–89]. Individuals living under difficult circumstances might thus be expected to display elevated thyroid hormone levels, an accelerated metabolism as well as a shorter lifespan. A Brazilian cohort study found that an underactive thyroid is less prevalent in lower income classes and in those with less years of education [90]. In elderly UK people, it was similarly found that subclinical **hyperthyroidism** was significantly associated with socioeconomic deprivation [91].

##### Energetic stress early in life

Assuming equal risks of extrinsic mortality, energetic stress may lead to decreased thyroid hormone levels. Decades of research on the ecology of human reproductive function have shown that differences in gestational and childhood environments alter the hypothalamic–pituitary–gonadal axis regulation and associated metabolic processes, as immunologically, nutritionally, or otherwise energetically stressed populations exhibit chronically lower ovarian steroid levels than more affluent population [92]. Similar processes might be at play in shaping diversity in thyroid function over the life course. For instance, **T3** levels are lower in babies born to anaemic and malnourished mothers [93], and women with low birth weight and smaller size at birth have an increased risk of developing an underactive thyroid in adulthood [94]. Lower **T4** levels were recorded in 61–70 year old British women bottle-fed or weaned early as compared to those who were breast-fed beyond their first year of life, suggesting an important role of infant nutrition and maternal thyroid hormones in breast milk for adulthood **HPT axis** regulation [95]. Another British cohort study found that among women aged 60–64 years, childhood weight gain from birth to puberty independently of height gain is positively associated with the presence of anti-TPO antibodies and **T4** use, which were used as indicators of thyroid dysfunction, whereas being overweight or obese at age 14 also correlated with positive anti-TPO antibodies [96]. **Hypothyroidism** furthermore clusters with other metabolic-endocrine disorders, such as cardiovascular disease, obesity, and polycystic ovary syndrome, which have all previously been linked to prenatal undernutrition and small gestational size followed by improved nutritional conditions during childhood [97, 98]. Further investigation is needed regarding the relationship between nutritional status in early life and adult thyroid function, as well as the role of epigenetic mechanisms in mediating these effects.

#### Acclimatization: short-term adjustments in thyroid function

Thyroid hormones might enable organisms to adjust to current changes in their environment and associated energetic conditions. Research suggests that extreme temperatures, infections, resource scarcity, as well as psychological or social stresses, alter the thyroid function in humans and other animals. For example, thyroid hormones are upregulated in male California sea lions during the breeding season, enhancing energetic investment into costly reproductive behaviour [99], whereas by contrast, fasting reduces **T4** levels in juvenile Northern Elephant Seals [100]. Although the **HPT axis** is stimulated by some (anticipated) acute energy-demanding situations including cold or exercise, bodily states of overall negative energy balance such as chronic stress, inflammation, or fasting, suppress the **HPT axis**’ activity [39, 101]. Rather than conserving a constant set point and feedback control within the **HPT axis**, as expected based on the concept of **homeostasis** under situations of strain and stress, the **HPT axis** acts as a flexible system that adjusts metabolism efficiently in anticipation of energetic challenges, which aligns with the model of adaptive **allostasis** [15, 101, 102]. In this context, **allostasis** is a dynamic stress reaction that maintains stability in the internal milieu through change, complementing homeostatic processes [101]. Across animal species, downregulated thyroid function might be an adaptive response to sustained energetic stress, Sea otters under resource stress for example experience **T3** levels that are reduced by 12% on average [103]. The expression of the deiodinase enzymes, which mediates the peripheral conversion of **T4** to **T3**, may also be suppressed as a result of the activation of the stress response [49, 104]. This effect is most likely mediated by glucocorticoids and inflammation [49], changing the expression of these enzymes and thereby creating a flexible system that can accommodate ecological challenges. In fasting juvenile Northern Elephant Seals for example, cortisol levels are inversely associated with free **T3** levels [100]. This suggests that lowered thyroid function preserves limited resources in the face of sustained energetic stress. By contrast, in situations where substantial increases in energy demands are anticipated and energetic resources are abundant, such as during pregnancy or in psychosocial stressful situations, active thyroid hormone production is upregulated [101]. In this context, elevated **T3** levels in patients with combat-related post-traumatic stress disorder [105] can be reinterpreted as an exacerbation of an otherwise adaptive response to an anticipated energy-demanding situation such as conflict. Although both homeostasis and **allostasis** are critical to thyroid function, the existence of allostatic adaptations in response to anticipated energetic stresses further challenge the appropriateness of using reference intervals created based on the assumption of homeostatic regulation only [101]. Such reference intervals may not be appropriate to accurately diagnose thyroid dysfunction in a physiological system that is in flux.

**Figure 3.**
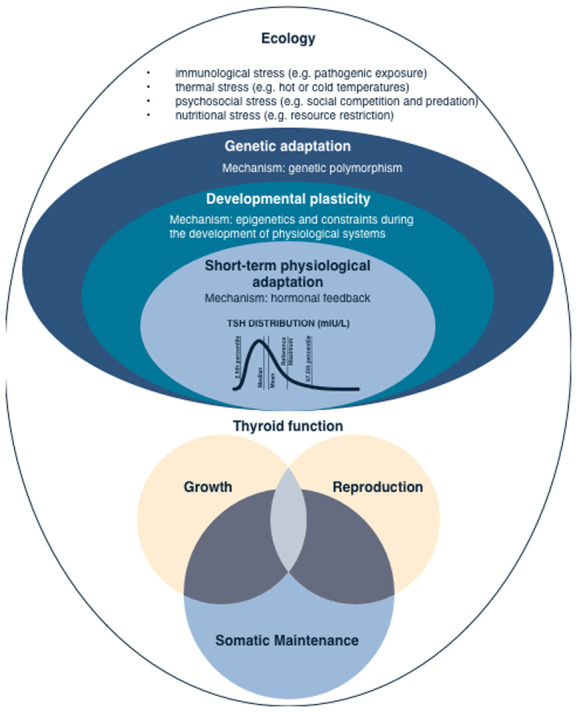
An evolutionary ecological framework for variation in thyroid function. Energetic challenges due to various ecological factors will induce alterations in thyroid function regulation depending on the life-history stage. Variation in thyroid function can be understood at multiple levels: genetic, developmental, and acclimatization. Given evolutionary constraints and ecological contexts, thyroid function plays a role in regulating the energetic trade-offs between the functions of reproduction, growth, and somatic maintenance, which includes immunity and basal metabolic rate. This evolutionary ecology framework can contribute to re-evaluate biomedically accepted cut-offs that are considered pathological. Yet, currently not enough is known about normal context-dependent patterns of variation, a prerequisite to understand the aetiology and onset of thyroid dysfunction.

Although research in humans is limited, thyroid hormones appear to be key modulators of the optimal investment of energy into the competing physiological functions of growth, reproduction, and somatic maintenance in other animals [106]. Using an evolutionary framework, we suggest that energetic challenges resulting from ecological pressures such as pathogenic exposure or cold temperatures mobilize genetic, developmental, and short-term adaptations, which may lead to significant natural variation in thyroid hormone levels (Fig. 3).

### 4. Evolutionary perspectives on thyroid pathologies

#### Evolutionary approaches to health and diseases

The field of Evolutionary Medicine and Public Health [86, 107–109] proposes a novel framework for disease aetiology that goes beyond understanding the proximate causes of illness only. Instead of narrowly considering the mechanisms responsible for thyroid disease, i.e. the immediate molecular, physiological, and anatomical causes, an evolutionary medicine approach considers the broader evolutionary, sociocultural, and developmental context from which these vulnerabilities to pathology emerged. Below we discuss the causes of thyroid disorders in light of this framework, focusing on four aetiologies of thyroid disease; (i) mismatches in diet composition and lifestyle; (ii) maternal–foetal conflict and the female preponderance in thyroid disease; (iii) host–pathogen co-evolution and emerging infections; and (iv) exacerbated defence mechanisms.

#### Evolutionary mismatch and micronutrient deficiencies

Low levels of micronutrients are often implicated in the aetiology thyroid disorders, suggesting that diet is an important pre-disposing factor in the development of thyroid disease [110]. A third of the world population lacks adequate iron intake, impairing the function of the haem-dependent **TPO-enzyme** involved in thyroid hormone synthesis [111], 15% of the world population is deficient in selenium, an integral component of the **deiodinase enzymes** converting **T4** into **T3** [111], and two billion people are iodine deficient, including 50% of continental Europe [112]. While seasonal and geographical variation in the availability of micronutrients, such as iodine, probably characterized the environment in which our species evolved [113], the widespread prevalence of iodine deficiency could be an evolutionary novel phenomenon [22, 114], resulting from the agricultural transition, the industrialization of food production, and the dietary shifts that accompanied these events [22, 114].

Although our physiology has evolved to be flexible and quickly adapt to new ecologies, cultural changes associated with the Neolithic agricultural transition and the more recent industrialization and urbanization happened so rapidly that our physiology struggles to catch up [115]. Certain food items rich in iodine such as seaweed, sea food, dark green leafy vegetables, egg yolk, and seeds, were probably more common in our ancestral diets [22]. In seemingly iodine-depleted regions, such as the Congo basin, the consumption of iodine-rich aquatic plants has even been reported in non-human primates [116]. However, an increased consumption of cereal grains, legumes, and tubers associated with the adoption of agriculture may have heightened our ingestion of substances that interfere with our body’s ability to utilize dietary iodine effectively, also known as **goitrogens** [117]. As a carbohydrate-rich diet increases **T3** levels, Kopp [114] has furthermore proposed that adopting a cereal based diet might contribute to an increase in iodine requirement in populations relying on agriculture. Increased flooding and soil erosion associated with intensified agriculture can deplete iodine in the soil, which causes the crops grown in such areas to be much lower in iodine content [112]. Despite the important contributions of globalization and iodized salt in decreasing iodine deficiency and its severe health consequences, as people move towards a more industrialized, Western diet, their intake of iodine decreases yet again due to the lack of iodized salt in processed and fast foods [118].

Universal iodine supplementation may seem like the easiest solution, but the effect of iodine supplementation on the development of thyroid dysfunction follows a U-shaped curve—both too little or too much iodine can contribute to an increased prevalence [2]—which is why variation in local geography, cultural practices regarding food, and evolutionary history should be taken into account when designing public health interventions. Similarly, other novel environmental exposures that might contribute to thyroid disease, such as air pollution and environmental pollutants, as well as night shift working and other risk factors associated with an urbanized, modern lifestyle deserve further consideration from biological anthropologists and public health practitioners alike [119–121]. Smoking, for example, may increase **T4**. Indeed lower **TSH** levels and **hyperthyroidism** are more prevalent amongst smokers [122], whereas obesity, by increasing leptin levels, might contribute to the development of thyroid autoimmunity and **hypothyroidism** [123].

#### Sex disparities in thyroid disease and the maternal-offspring conflict

Although according to some research, parity itself does not put women at risk of developing thyroid dysfunction later on [124], women are especially vulnerable to developing thyroid conditions around reproductive transitions such as pregnancy; in the US 15.5% of pregnant women are hypothyroid [125] and 5–8% of iodine-sufficient women experience transient thyroid dysfunction postpartum [22, 126]. In this context reinterpreting the meaning of reproductive disease is necessary to move the women’s health agenda beyond studying the pathologies of reproductive organs alone [127].

There might have been significant selection pressure throughout human evolutionary history to adequately manage the maternal–offspring conflict over thyroid hormones and iodine access during pregnancy, which may predispose women to develop thyroid disease. Evolutionary biologists consider gestation as a period of both mutual and competing interests because of the dissimilar ‘goals’ of mother and foetus [109, 128, 129]. Whereas the offspring seeks to maximize maternal investment into thyroid hormone production during pregnancy to enhance its own long-term fitness and iodine access, the maternal body has evolved to optimize thyroid hormone allocation in a way that does not irreversibly harm her health, iodine stores and future fertility [129]. Pregnancy increases the mother’s daily iodine requirement due to enhanced renal excretion and an increase in thyroid hormone synthesis by 50–75% [22]. Although the thyroid is the first endocrine gland developing in the offspring, it only becomes active after the 12th week of gestation and the progeny continues to rely on maternal thyroid hormone throughout pregnancy and lactation to gain access to iodine [101, 130]. Therefore, the embryo depends on the mother’s thyroid hormone production during this critical early period of neural tissues differentiation early in pregnancy [7, 129], thereby inevitably also relying on the mother’s iodine status as a result of her diet. Iodine deficiency during pregnancy may cause miscarriage or brain damage in the offspring [22], whereas at the same time iodine-deficient mothers risk developing a **goitre** leading to thyroid dysfunction and even infertility later on [109, 129].

Understanding changes in thyroid function regulation during pregnancy may also shed new light on the aetiology of thyroid

that a maternal–offspring conflict may take place over thyroid hormones and iodine access during pregnancy, resulting in a physiological tug-of-war [129, 131]. Boddy *et al.* (2015) have proposed that foetal cells preferentially infiltrate maternal tissues important in resource allocation [131]. In the context of the maternal–offspring conflict over iodine, we suggest that in addition to using enhanced thyroid metabolism as a means to upregulate maternal heat production postpartum, foetal cells may also enhance thyroid hormone production during pregnancy and lactation to enhance iodine access during gestation and iodine transfer via the mother’s milk. However, recognition of foetal cells by the maternal immune system could cause autoimmune reaction against thyroidal tissue and the high prevalence of postpartum thyroiditis [109]. Accordingly, foetal **microchimerisms** are found more frequently in the thyroids of -patients than in healthy volunteers or nulliparous women [132, 133] and may also be preferentially located in thyroid neoplasms compared to healthy thyroids [134]. Therefore, **microchimerisms** may be one mechanism underlying the preponderance of thyroid disease in women.

Oestrogen is also closely linked to maternal thyroid function during gestation, as studies of combined oral contraceptive users show that using hormonal contraception, which in certain ways mimics pregnancy conditions, increases pituitary sensitivity towards thyrotropin-releasing hormone (TRH) and is associated with higher thyroid hormone levels [22]. Sievert (2017) has suggested that due to antagonistic pleiotropy the adaptive connection between oestrogen and thyroid function during pregnancy may be deleterious when oestrogen levels fall during menopause, contributing to the sex disparity in thyroid disease postmenopausally [22]. Yet, a large cohort study in oral contraception users suggests that oestrogen might actually be protective against developing thyroid disease whilst taking contraceptives [135], whereas the effect of contraception discontinuation remains under-researched. It remains to be investigated what other adaptations enabling the manipulation of the maternal thyroid during pregnancy might also be proximate mechanisms by which thyroid disease develops during other reproductive transitions such as menopause [22].

Another proposed mechanism for the maternal–offspring conflict over **HPT axis** regulation is based on the structural similarity between human Chorionic Gonadotropin (hCG), the ‘pregnancy hormone’ secreted by the embryo during the first few weeks of gestation, and TSH [136, 137]. Due to its chemical similarity to **TSH**, hCG is able to attach to **TSH**-receptors on the thyroid, stimulating the growth of the gland and **T4** and **T3** production during gestation [129, 138, 139]. The offspring in turn uses the placental deiodinase 3 enzyme to convert any excess of maternal thyroid hormone into its inactive forms, such as reverse **T3** (rT3) [129]. This process, in which hCG acts as a thyroid stimulator, has been viewed to serve embryonic interests at the cost of maternal interests under condition of iodine deficiency [129]. Forbes (2014) argues that when iodine is plentiful, the correlates of surplus thyroid hormone production (e.g. rT3) might be proximate triggers for pregnancy sickness [129, 140], although this remains to be tested. More generally, because the manipulation of the maternal thyroid by hCG during pregnancy is produced outside the maternal thyroid control circuit, thyroid function is prone to dysregulation. In the context of Flaxman and Sherman’s seminal work on nausea and vomiting during pregnancy in relation to food aversions and cravings, it would also be relevant to see whether goitrogenic foods, which interfere with thyroid metabolism, are considered more aversive during early gestation in societies that are relatively iodine-depleted, whereas iodine-rich foods are being craved [141].

#### Old Friends, emerging infections, and

Complex interactions exist between pathogen exposure and risk. The Old Friend hypothesis proposes that some contemporary environments deprive humans of the input from microorganisms, such as helminthic parasites, that induce important regulatory pathways involved in immunotolerance during development [142]. The involvement of ‘Old Friends’ in these pathways is the result of a symbiotic co-evolution with these microorganisms throughout mammalian evolutionary history. Helminths have as a result acquired the capacity to manipulate the host immune system for their own benefit by inducing a modified Th2-type immune response and inducing regulatory T- and B-cells to down regulate the host’s inflammatory responses [142, 143]. Some modern lifestyles, lacking this helminth-induced immunoregulation, might therefore predispose to disorders associated with excessive inflammation, including autoimmunity [142, 144]. In this line, a study in mice shows that prior infection with the helminth *Schistosoma mansoni* suppresses inflammatory Th1-type immune responses against the TSH-receptor, thereby preventing Graves’ disease development [145]. Reintroducing coevolved microbiota has been used as a treatment for some autoimmune diseases such as multiple sclerosis, where helminth therapy shows some beneficial effects, mostly by reducing inflammatory cytokine levels [143]. However, in the context of Graves’ disease in mice, helminth therapy is only effective *before* the aberrant immune response against the TSH-receptor has developed; later introduction of helminths was not effective [145]. Further studies are necessary to confirm whether helminth therapy, preferably in the form of helminth-derived immunomodulatory molecules rather than living parasites, may have a beneficial effect in preventing or treating s in humans, as the safety of using live parasites is still under investigation [146].

Persistent infections of the thyroid and the manipulation of the host’s immune system by microbial organisms and viruses can also contribute to [147–149]. For Hashimoto’s thyroiditis, viruses such as herpes simplex, rubella, mumps, and Epstein-Barr might play a role in disease development, whereas retroviruses such as HIV are associated with Graves’ disease [147, 149]. Through either **bystander activation** or **molecular mimicry**, the inflammatory cellular immune response to a viral infection can cause the activation of autoreactive immune cells that target thyroid antigens themselves and persist even after the infection has cleared [150].

The spread of the novel coronavirus could pose a novel environmental trigger contributing to increased thyroid dysfunction post-pancemic. In 61 survivors of the SARS outbreak in the early 2000s, 3% had transient subclinical thyrotoxicosis and nearly 7% had underactive thyroid following recovery [151]. One of the pathological organ changes described after the SARS epidemic was increased thyroid follicular cell death [152], which in combination with enhanced cytokine production and systematic inflammation, could lead to the activation of autoreactive immune cells against thyroid antigens and a breakdown in tolerance. Similarly, in the current COVID-19 pandemic significant alterations in thyroid function have been noted: compared to patients suffering from non-COVID-19 pneumonia, COVID-19 patients have significantly decreased TSH and total **T3** levels depending on the severity of the disease [153]. Furthermore, several case reports suggest that in some survivors of the disease, SARS-COV-2 may act as a trigger of for autoimmune **hyperthyroidism** (Graves) and thyrotoxicosis [154–157], even if initial disease course was mild [157].

#### Exacerbation of adaptive responses

In some instances, pathology may result from an exacerbation of a normally adaptive response [107, 158]. An example is the phenomenon of ‘non-thyroidal illness syndrome’ (NTIS), occurring in 60–70% of critically ill patients [38], which is characterized by extremely low levels of thyroid hormones despite TSH being initially unaltered [31]. Patients admitted to the intensive care unit for various acute illnesses have higher reverse **T3** (rT3) levels due to the inactivation of **T3** by deiodinase enzymes, and disease severity correlates with higher concentrations of rT3 [159], suggesting that deiodinase enzyme expression is altered [101]. Patients with low **T3** require longer ventilation and experience higher mortality rates [101], and low serum **T3** have also been recorded in 96% of hospitalized patients with SARS-CoV-2 infection [149, 160].

Despite several clinical trials attempting to correct abnormal thyroid function in NTIS using thyroid hormone replacement, there is not enough improvement in patient outcomes to justify the use of such therapeutic approaches[161]. The evolutionary ecology framework proposed here, with its focus on energy balance, suggests that improving energetic conditions rather than thyroid hormone supplementation would benefit patients with NTIS, which is a contribution from evolutionary medicine to clinical medicine. Indeed, a recent study shows that high caloric exposure can attenuate the decrease of **T3**, aiding patient recovery [162]. NTIS can therefore be reinterpreted as an exacerbated adaptive response to illness in which thyroid function is downregulated to lower energetic requirements of non-vital functions in the face of chronic negative energy balance during illness [53, 163]. To shed light on the extreme ends of normal variation in thyroid function, characterizing the role of different types of stress on thyroid function and **T4** to **T3** conversion by deiodinases is required.

## CONCLUSION

This review sought to challenge the idea that all variation in thyroid function is best interpreted as pathological. We have advocated for an evolutionary perspective to shed new light on normal patterns of variation across populations, individuals and over the lifespan, framing diversity in thyroid function as an evolved response allowing organisms to adapt their metabolism to the energetic requirements of their ecology (Fig. 3). The insight that the thyroid system evolved in response to various species-specific environmental challenges to serve multiple functions can help draw a holistic framework integrating multiple ecological factors to comprehend normal patterns of variation, and helps identify a number of outstanding questions (Box 5). To that end, using big data and digital health apps might help us access untapped sources of variation to develop a deeper insight into the associations between an individual’s life-history and ecology on the one hand and changes in thyroid function and symptomatology on the other hand. By creating a better understanding of the natural variation in thyroid function, we can start providing the more personally adjusted care that patients deserve by giving greater attention to the underlying causes of thyroid dysfunction rather than focusing on treating patients within constructed reference values.

Box 5. **Outstanding questions**

**Understanding natural variation:**

- What are the reference ranges for thyroid function in natural fertility, subsistence populations living in different ecologies?
- How does ageing affect thyroid function in populations not living a Westernized industrial lifestyle?
- Why is there variation in cretinism subtypes across different geographical regions?

**Genetic adaptation:**

- Are there substantial differences in thyroid function and genetic background between populations consuming a shore-based diet and those that live in iodine-depleted regions?
- What are the genes responsible for differences in thyroid function between humans and other primates?

**Developmental plasticity:**

- How does maternal thyroid function during pregnancy affect the epigenetic regulation of the offspring’s thyroid function?
- How does breastfeeding and infant nutrition affect thyroid function in childhood, adulthood, and post-reproductive life?
- Does psycho-socio-economic adversity in childhood influence adult thyroid function?

**Acclimatization:**

- How do different types of ecological stressors (e.g. infection, undernutrition, social competition) affect the expression of the deiodinase enzymes that locally convert T4 to T3?
- How does being in COVID-19 pandemic lockdown affect thyroid function?

**Understanding thyroid disease using an evolutionary medicine approach:**

- Why is there more in populations that in the past were chronically iodine-depleted but are now (over)supplemented with the micronutrient?
- What is the disease burden of in populations living in different pathogen ecologies?
- Do the proximate mechanisms that link pregnancy to changes in thyroid function play a role in the aetiology of thyroid disease during reproductive transitions?

## Supplementary data

Supplementary data are available at *EMPH* online.

## Supplementary Material

eoaa043_Supplementary_Data

## Glossary

**.** Autoimmune thyroid diseases include all autoimmune reactions against thyroid autoantigens that may lead to destruction or hyperstimulation of the thyroid gland. Two examples of are Graves' disease and Hashimoto's.

**Allostasis.** A concept suggesting that adjusting physiological systems to changing environments adaptively requires a flexible anticipation of energetic challenges and short-term alterations in feedback set points.

**Athyrosis.** Congenital absence of the thyroid. Can result in myxedematous cretenism if not corrected early.

**Autoantibodies.** Antibodies that target the body's own tissues are called autoantibodies. Three common autoantibodies relevant to the thyroid are those specific for the TPO-enzyme, TGB or the TSH-receptor.

**Bystander effect.** During an inflammatory immune response in a certain tissue due to pathogen invasion, autoreactive immune cells that have escaped the selection process during development can be accidentally activated, causing the development of autoimmunity.

**Cretinism.** Congenital iodine deficiency syndrome. Usually, a distinction is made between two types, endemic cretinism and **myxedematous cretinism** (see below).

**Convergent evolution.** When different species or taxa evolve a similar feature or function independently at different time points in their evolutionary history.

**Endothermy.** The ability to self-regulate body temperature.

**Exaptation.** A co-option of an anatomical structure, a certain behavioural trait or a physiological system during the evolutionary history of a species to serve a novel function.

**Goitre.** A swelling of the neck due to an enlarged thyroid gland.

**Hyperthyroidism.** An overactive thyroid characterized by high T4 levels and low TSH levels.

**Hypothyroidism.** An underactive thyroid characterized by low T4 levels and high TSH levels.

**HPT axis.** The hypothalamic–pituitary–thyroid axis (see Fig. 1).

**Life-history theory.** A framework in evolutionary biology aimed at understanding diversity in life-histories between species and individuals.

**Maternal hypothyroxinaemia.** Low T4 levels in the mother during gestation, which can cause cretinism in the offspring.

**Microchimerisms.** The bidirectional exchange of maternal and foetal cells during gestation. When these cells are not adequately cleared after parturition, they are a predisposing factor for the development of autoimmunity as they may cause the body to launch an immune response against the foreign invading cells. Some research suggests that foetal cells may preferentially travel to the maternal thyroid amongst other tissues.

**Molecular mimicry.** Pathogens have evolved the capacity to escape recognition by the host immune system by mimicking the host's own bodily molecules on their surface. However, this means that an immune response against these molecules may have a cross-over effect with the host's own tissues, which can result in the development of aberrant immune responses and autoimmune disease after the infection.

**Myxedematous cretinism.** Results from severe iodine deficiency in late pregnancy or the neonatal period, causing mental retardation, short stature, goitre, and hypothyroidism. Can also be caused by the underdevelopment or atrophy of the thyroid in the offspring.

**Neurologic cretinism.** Results from maternal iodine deficiency or hypothyroidism during pregnancy, leading to intellectual disability, deaf mutism, spasticity, and disturbances in gait in the offspring. However, the offspring has adequate thyroid function postnatally.

**Plasticity.** The evolved capacity of an organism to adjust its physiology, development, and behaviour to variable environments in a way that maximizes its overall fitness.

**T3.** Triiodothyronine (T3) is the more metabolically active type of thyroid hormone which is taken up by cells but has a shorter half-life in the circulation compared to T4.

**T4.** Thyroxine (T4) is the less active variant of thyroid hormone but binds to thyroid hormone binding proteins with higher affinity, and therefore has a longer half-life in the circulation.

**Thyroglobulin (TGB).** Intermediate/precursor product of thyroid hormone synthesis, can be stored for longer periods of time in the thyroid.

**TPO(-enzyme).** Thyroid hormones are synthesized through the iodination of the amino acid tyrosine by the enzyme thyroid peroxidase (TPO).
