# Supplementary material for: Reinterpreting patterns of variation in human thyroid function: An evolutionary ecology perspective
Source: Evol Med Public Health. 2020 Nov 10;9(1):93–112. doi: 10.1093/emph/eoaa043 (PMC8454515; doi:10.1093/emph/eoaa043)
Supplement: eoaa043_Supplementary_Data [file eoaa043_Supplementary_Data.pdf]

Supplementary Table 1: Female TSH reference intervals (2.5th and 97.5th percentiles) published from 2000 according to reproductive life stage

Methodology and references systematic search female TSH reference intervals

The figures attached as a supplementary to the review "Understanding Patterns of Variation in Human Thyroid Function: An Evolutionary Perspective" as well as Figure 2 in the main text of the article display a systematic search of papers published since the year 2000 stating a female specific TSH reference interval mentioning the 2.5th and 97.5th percentile and the median value of the population. Search terms used: "reference interval OR reference range" "TSH OR thyrotropin" "female OR women" for Google Scholar and "((((female) OR (women))) AND (((TSH) OR (thyrotropin)))) AND (((reference range) OR (reference interval)))" for PubMed. Selection criteria for inclusion of the studies were that female specific TSH values were given in two decimals in the unit mIU/l and that the median (50th percentile) and 2.5-97.5 percentile range was stated in the article. Occasionally articles stated the mean rather than the median, and although these articles are not used for the creation of the figures, they are displayed in the tables below, indicated with an * symbol. We looked at articles published from the year 2000 onwards to reflect the latest advances in laboratory techniques. Only English language articles with full text available on the internet and accessible through the University of Oxford's institutional subscriptions were considered for the purpose of this systematic review. The first 150 articles that came up on Google Scholar and PubMed were screened and only primary studies were downloaded and processed in Mendeley. During the abstract screening publications that were not primary studies on TSH reference intervals were excluded. After the first screening 89 studies found on Google Scholar were assessed for full text review. For PubMed, after removal of duplicates that were already downloaded from the Google Scholar search, 50 studies were considered for full-text review. For papers that reported TSH values for an extensive range of ages (marked with a [1] symbol) examples were randomly chosen, and for gestational week specific data we selected a data point in five-week intervals to be displayed and added the exact week was added under additional comments. Where both the total population TSH reference range and that of a reference/disease-free population was reported, we chose to display the population selected to exclude individuals with thyroid disease in order to reflect the 'healthy', normal range of variation in TSH levels. We also excluded measurements that were based on less than thirty participants in a specific category. In figure 2, we only included TSH reference intervals that were published from 2017 onwards for the sake of space, however in the tables presented in this supplementary file reference intervals since the year 2000 are included. We organised the female TSH reference intervals published in the last twenty years during childhood (0-13 years), the reproductive years (non-pregnant women aged 13-49 years), pregnancy trimesters, and post-reproductive life stages (50+ years). Below there is an explanation of the 60 studies that were excluded after full text assessment and reason of exclusion. Please note that this exercise is not a systematic review but instead a systematic search of the literature for the purpose of displaying the geographical variation and the effect of reproductive life phase on TSH reference intervals across the world.

Supplementary Table 1: Female TSH reference intervals (2.5th and 97.5th percentiles) published from 2000 according to reproductive life stage

OUTCOME SYSTEMATIC SEARCH FEMALE TSH REFERENCE INTERVAL

Total number of papers screened: 300
Included studies for full text assessment: 126
Excluded papers after abstract screening: 174
Included studies for tables and figures: 66 studies
Excluded studies after full text assessment: 60 studies

- Studies that did not differentiate between male and female reference intervals: 23 (Kawahara and Yokoya, 2002; Singh, Parvin and Gronowski, 2003; Völzke *et al*., 2005; Dhatt, Griffin and Agarwal, 2006; Kratzsch *et al*., 2008; Kapelari *et al*., 2008; Boucai and Surks, 2009; Takeda *et al*., 2009; Rosario *et al*., 2010; Boucai, Hollowell and Surks, 2011; Schalin-Jäntti *et al*., 2011; Chan, Iu and Shek, 2011; Mutlu *et al*., 2012; Chaler *et al*., 2012; Waring *et al*., 2012; Fontes *et al*., 2013; Rosario and Calsolari, 2014; Mirjanic-Azaric *et al*., 2017; Valdés *et al*., 2017; Ishaku, Shabbal and Isichei, 2017; Jeon *et al*., 2017; Jayasuriya *et al*., 2018; Wang *et al*., 2019)
- Studies that did not give 2.5th-97th reference intervals for TSH: 35 (Knudsen *et al*., 2000; Eltom *et al*., 2000; Price *et al*., 2001; Yeo *et al*., 2001; Soldin *et al*., 2004; Kurioka, Takahashi and Miyazaki, 2005; Zarghami, Rohbani-Noubar and Khosrowbeygi, 2005; O'Leary *et al*., 2006; Soldin, Soldin and Sastoque, 2007; Jang *et al*., 2008; Lambert-Messerlian *et al*., 2008; Marwaha *et al*., 2008, 2010, 2013; Ahmed *et al*., 2009; Adeniran, Okolo and Onyiriuka, 2010; Mansourian *et al*., 2010; Inal *et al*., 2010; Su *et al*., 2011; Yoshihara *et al*., 2011; Raverot *et al*., 2012; Roelfsema and Veldhuis, 2013; Springer, Bartos and Zima, 2014; Mankar, Sahasrabuddhe and Pitale, 2016; Almomin, Mansour and Sharief, 2016; Omuse *et al*., 2016; Cai *et al*., 2016; Elhaj *et al*., 2016; Hoermann *et al*., 2016; Mirjanic-Azaric *et al*., 2017; Hickman *et al*., 2017; Musa *et al*., 2018; Kaluarachchi *et al*., 2019; Zhang *et al*., 2019; Donovan *et al*., 2019)
- Studies that give no new reference interval: 11 (Knudsen *et al*., 2000; Moncayo, Dapunt and Moncayo, 2007; Ross *et al*., 2009; De Moura Souza and Sichieri, 2011; Hadlow *et al*., 2013; Velayutham, Selvan and Unnikrishnan, 2015; Langén *et al*., 2017; Murillo-Llorente *et al*., 2017; Kang, Hwang and Chung, 2018; Lee *et al*., 2018; Nazeri *et al*., 2018)
- Studies that report in a different unit: 1 (Adeniran, Okolo and Onyiriuka, 2010)
- Studies that do not specify age range clearly: 2 (Wang *et al*., 2017; Clerico *et al*., 2019).

Supplementary Table 1: Female TSH reference intervals (2.5th and 97.5th percentiles) published from 2000 according to reproductive life stage

TABLE 1: RESULTS FROM GOOGLE SCHOLAR SEARCH TO BE INCLUDED IN FIGURE 2 AND SUPPLEMENTARY FIGURES

Paper authors	Reference number	Childhood	Adulthood (not pregnant)	Pregnancy trimester			Post-repro-ductive	Nr of subjects	Ethnicity	Selection participants	Assay manu-facturer	Any comments, including (gesta-tional) age.
				1st	2nd	3rd						
(Abraham *et al*., 2009)	1		1.8 (1.2-4.7)					*n*=44	Indian	Excluded those with previous or present thyroid diseases or diabetes	Diagnostic Product Corporation	20-29 years
			1.8 (1.1-5.1)					*n*=120				30-39 years
			2.0 (1.1-4.1)					*n*=159				40-49 years
							2.0 (1.1-4.1)	*n*=119				50-59 years
							2.1 (1.2-5.4)	*n*=48				60-69 years
(Aldrimer *et al*., 2012)	2		(0.43-3.35)					*n*=119	Swedish	Excluded those with chronic diseases or infection	Abbott	13-18 years
(Aoki *et al*., 2007)	3		1.37 (0.38-4.69)					*n*= 1607	American	Total sample	Immuno-assay Techniques (manu-facturer not specified)	12-49 years
							1.67 (0.24-7.16)	*n*=583				50-79 years
							2.09 (0.31-9.39)	*n*=130				>80 years
(Ashoor *et al*., 2010)[1]	4			1.05 (0.08-3.09)	1.19 (0.13-3.34)			*n*= 3125	White British	Excluded those with complicated pregnancies and women with known thyroid disease or autoanti-bodies	Siemens	11th and 13th week BMI<25
				1.08 (0.09-3.15)	1.23 (0.14-3.41)							11th and 13th week BMI>25
				0.71 (0.01-2.49)	0.83 (0.03-2.71)			*n*=759	Black British			11th and 13th week BMI<25
				0.73 (0.01-2.54)	0.86 (0.04-2.77)							11th and 13th week BMI>25
(Bailey *et al*., 2013)	5	2.31* (0.73-4.77)						*n*=139	Canadian	Excluded those with metabolic, chronic, or acute illnesses	Abbott	4 days <6 months
		1.98* (0.70-4.17)						*n*=640				6 months<14 years
			1.51* (0.47-3.41)					*n*=259				14<19 years
(Bliddal *et al*., 2014)	6			1.38* (0.39-3.42)				*n*=44	Danish	Excluded those with complicated pregnancies, twin pregnancies, thyroid auto-antibodies, or known thyroid disease	Roche	10-15th week of gestation
					1.42* (0.40-5.05)			*n*=86				15-20th week of gestation
					1.45* (0.54-3.90)			*n*=92				20-25th week of gestation
					1.34* (0.51-3.52)			*n*=86				25-30th week of gestation
						1.37* (0.53-3.57)		*n*=90				30-35th week of gestation
						1.72* (0.71-4.15)		*n*=79				35-40th week of gestation
(Bliddal *et al*., 2015)	7			(0.1-3.6)				*n*=455	Danish	Excluded those with twin pregnancies, known thyroid disease or diabetes	Roche	Mean gestational age 10.8 weeks
					(0.3-3.7)			*n*=126				Mean gestational age 12.7 weeks

Supplementary Table 1: Female TSH reference intervals (2.5th and 97.5th percentiles) published from 2000 according to reproductive life stage

Paper authors	Reference number	Childhood	Adulthood (not pregnant)	Pregnancy 1st trimester	Pregnancy 2nd trimester	Pregnancy 3rd trimester	Post-reproductive	Nr of subjects	Ethnicity	Selection participants	Assay manufacturer	Any comments, including (gestational) age.
(Dashe *et al.*, 2005)[1]	8			1.36 (0.23-4.94)				*n*=368	American	Excluded were triplet and quadruplet pregnancies.	Diagnostic Products Corporation	6th week gestational age
				0.76 (0.01-3.65)				*n*=888				11th week of gestation
					0.92 (0.04-2.74)			*n*=456				16th week of gestation
					1.21 (0.28-3.04)			*n*=317				21st week of gestation
					1.07 (0.20-2.89)			*n*=237				26th week of gestation
						1.06 (0.23-2.81)		*n*=172				31st week of gestation
						1.31 (0.33-4.59)		*n*=144				36th week of gestation
						1.68 (0.38-5.43)		*n*=312				≥40th week of gestation
(Dhatt *et al.*, 2006)[1]	9		1.32* (0.3-4.32)					Not speci-fied	UAE Arab	Population free of thyroid disease	Abbott	6-38 weeks, median 16 weeks for whole study cohort.
				0.71* (0.06-8.3)				*n*=97				
					1.04* (0.17-5.9)			*n*=122				
						1.20* (0.21-6.9)		*n*=79				
(Ehrenkranz *et al.*, 2015)	10	(0.56-5.13)						*n*=27,325	American	Excluded those with clinical thyroid disease	Abbott	1-20 years
			(0.53-4.93)					*n*= 74,288				21-40 years
							(0.54-5.34)	*n*= 61,032				41-60 years
							(0.52-6.20)	*n*= 31,930				61-80 years
							(0.54-6.07)	*n*= 6,637				>80 years
(Gilbert *et al.*, 2008)	11			(0.02-2.16)				*n*=515	Australian	Whole reference group	Abbott	11th week
					(0.06-2.54)			*n*=114				13th week
(Lombardo Grifol *et al.*, 2013)	24			(0.48-3.80)				*n*=140	Spanish	Excluded those with thyroid or other endocrine diseases and complicated pregnancies	Siemens	8-13th week of gestation, mother aged 16-30
				(0.50-3.46)				*n*=79				8-13th week of gestation, mother aged 30-43
(Guan *et al.*, 2008)	12		1.32* (0.04-7.31)					*n*= 2827	Chinese	Excluded those that were pregnant, breast-feeding or taken oral contraception	Diagnostic Products Corporation	14-≥60 years

Supplementary Table 1: Female TSH reference intervals (2.5^{th} and 97.5^{th} percentiles) published from 2000 according to reproductive life stage

Paper authors	Reference number	Childhood	Adulthood (not pregnant)	Pregnancy 1st trimester	Pregnancy 2nd trimester	Pregnancy 3rd trimester	Post-reproductive	Nr of subjects	Ethnicity	Selection participants	Assay manufacturer	Any comments, including (gestational) age.
(Haddow *et al*., 2004)	13			0.94 (0.08-2.71)				*n*=1005	American	Whole sample	Diagnostic Products Corporation	8-13th week of gestation
					1.22 (0.39-2.70)			*n*=1005				15-21st week of gestation
(Hollowell *et al*., 2002)	14		1.25 (0.40-3.65)					*n*= 9310 (does not specify nr of subject per age category)	American	Excluded those who are pregnant, taking thyroid altering medications or with measurable thyroid antibodies or chemical evidence of hypothyroidism or hyperthyroidism	Nichols Institute Diagnostics	12-19 years
			1.26 (0.38-3.88)									20-29 years
			1.41 (0.33-4.71)									30-39 years
			1.59 (0.60-5.77)									40-49 years
							1.70 (0.52-10.74)					50-59 years
							1.99 (0.45-11.64)					60-69 years
							2.00 (0.46-12.97)					70-79 years
							2.10 (0.30-10.79)					≥80 years
(Ittermann *et al*., 2015)	15		(0.57-3.46)					*n*=617	German	Reference population free of thyroid disease and auto-antibodies	Siemens	Median age 51 years
(Jebasingh *et al*., 2016)	16			1.07 (0.21-1.82)				*n*=109	Indian	Excluded those with hyperemesis gravid arum, past/family history thyroid or connective tissue disease or thyroid-function altering medication..	Siemens	Mean gestational age 9.3 weeks
					1.23 (0.71-1.71)			*n*=148				Mean gestational age 20.8 weeks
						1.23 (0.70-1.93)		*n*=118				Mean gestational age 35 weeks
(Kahapola-Arachchige *et al*., 2012)	17	1.94 (0.65-3.82)						*n*=91	Australian	Excluded those with thyroid disease, taking interfering medication, children less than one year of age, and samples taken in the evening	Siemens	1-5 years
		1.90 (0.79-3.95)						*n*=334				5-10 years
			1.46 (0.51-3.56)					*n*= 4096				15-20-years
			1.46 (0.48-3.62)					*n*= 7264				25-30 years
			1.63 (0.53-3.99)					*n*= 8917				45-50 years
							1.71 (0.55-4.12)	*n*= 9053				50-55 years
							1.81 (0.47-4.53)	*n*= 4630				70-75 years
							1.80 (0.43-4.75)	*n*= 1663				85-90 years
							1.91 (0.52-5.59)	*n*=912				>90 years

Supplementary Table 1: Female TSH reference intervals (2.5^{th} and 97.5^{th} percentiles) published from 2000 according to reproductive life stage

Paper authors	Reference Number	Childhood	Adulthood (not pregnant)	Pregnancy 1st trimester	Pregnancy 2nd trimester	Pregnancy 3rd trimester	Post-reproductive	Nr of subjects	Ethnicity	Selection participants	Assay manufacturer	Any comments, including (gestational) age.
(Kim *et al*., 2018)	18	3.00 (1.56-4.41)						*n*=344	Korean	Excluded those with thyroid disease or auto-antibodies	BRAHMS AG	3-7 days after birth
			1.32 (0.03-4.24)					*n*=135				Mean enrolment age mother is 33 years. Does not specify exact gestational week
				1.62 (0.13-4.84)				*n*=143				
					1.59 (0.30-5.57)			*n*=139				
(Kratzsch *et al*., 2005)	19		1.42 (0.44-3.94)					*n*=174	German	Excluded those with endocrine, and metabolic disorders, taking thyroid-altering medication or underwent recent surgery	Roche	18-68 years
(Kut-luturk *et al*., 2014)	20		1.51 (0.22-4.53)					*n*=140	Turkish	Excluded those with thyroid disease or taking medications	Roche	30.1±11 years
(Kwon *et al*., 2017)	21	2.46 (0.56-7.50)						*n*=312	Korean	Excluded those with prior history of thyroid disease or taking medicines that could influence thyroid function	Roche	10-18 years
			2.30 (0.56-7.08)					*n*=507				19-29 years
			2.15 (0.48-6.67)					*n*= 510				30-39 years
			2.25 (0.70-12.60)					*n*=558				40-49 years
							2.64 (0.42-11.44)	*n*= 592				50-59 years
							2.39 (0.57-7.44)	*n*=650				60-69 years
							2.53 (0.44-6.89)	*n*=57				≥70 years
(La'ulu and Roberts, 2011)	22			0.89 (0.02-2.81)				*n*=424	Asian American	Excluded thyroid auto-antibody positive individuals	Abbott	10-13th week of gestation, age mothers is 15-46 years.
				0.82 (0.02-2.40)				*n*=506	Black American			
				1.02 (0.01-2.69)				*n*=511	Hispanic American			
				1.02 (0.11-2.69				*n*=731	White American			
(Liu *et al*., 2017)	23			1.43 (0.08-4.46)				*n*=312	Chinese	Excluded women with liver, kidney, or other system diseases, hyper-tension, diabetes and positive thyroid auto-antibodies	Abbott	4.57-12th week, median 8th weeks
					1.51 (0.05-3.71)			*n*=304				12.14-27th week, median 22 weeks
						1.97 (0.47-6.29)		*n*=331				27.14-40th week, median 36 weeks

Supplementary Table 1: Female TSH reference intervals (2.5^{th} and 97.5^{th} percentiles) published from 2000 according to reproductive life stage

Paper authors	Reference number	Childhood	Adulthood (not pregnant)	Pregnancy 1st trimester	Pregnancy 2nd trimester	Pregnancy 3rd trimester	Post-reproductive	Nr of subjects	Ethnicity	Selection participants	Assay manufacturer	Any comments, including (gestational) age.
(Maji *et al.*, 2014)	25			1.8 (0.25-3.35)				*n*=125	Indian	Excluded those with (family) history of thyroid disease, other chronic or bloodborn diseases, taking medication or complicated pregnancies, abnormal glucose, kidney or liver function test and positive thyroid auto-antibodies	Monobind Inc	<13th week of gestation
					1.84 (0.78-4.96)			*n*=151				13-27th week of gestation
						1.33 (0.64-1.99)		*n*=126				>28th week of gestation
(Männistö *et al.*, 2011)	26			1.26 (0.26-3.87)				*n*=269	Northern Finnish	Excluded those with thyroid disease or thyroid auto-antibodies	Abbott	2-6th week of gestation
				1.04 (0.09-3.34)				*n*=627				11th week of gestation
					1.17 (0.07-2.99)			*n*=325				13th week of gestation
					1.36 (0.35-3.32)			*n*=161				17-20th week of gestation
(Panesar, Li and Rogers, 2001)	27		1.17 (0.39-3.51)					*n*=63	Chinese	Excluded those with thyroid disease, hyperemesis gravidarum trophoblastic disease or pre-eclampsia	Chiron Diagnostics Corporation	20-54 years
				0.8 (0.03-2.30)				*n*=55				11th week of gestation
					0.87 (0.03-2.80)			*n*=66				15th week of gestation
					1.10 (0.03-3.10)			*n*=62				19th week of gestation
(Park *et al.*, 2018)	28	2.38 (0.56-7.50)						*n*=499	Korean	Excluded those with known thyroid disease, family history of thyroid dysfunction, or current pregnancy	Roche	10-19 years
			2.27 (0.62-6.39)					*n*=483				20-29 years
			2.24 (0.50-7.11)					*n*=490				30-39 years
			2.28 (0.55-11.86)					*n*=467				40-49 years
							2.64 (0.20-9.79)	*n*=471				50-59 years
							2.37 (0.56-7.87)	*n*=425				60-69 years
							2.42 (0.46-6.89)	*n*=48				>70 years

Supplementary Table 1: Female TSH reference intervals (2.5^{th} and 97.5^{th} percentiles) published from 2000 according to reproductive life stage

Paper authors	Reference number	Childhood	Adulthood (not pregnant)	Pregnancy 1st trimester	Pregnancy 2nd trimester	Pregnancy 3rd trimester	Post-reproductive	Nr of subjects	Ethnicity	Selection participants	Assay manufacturer	Any comments, including (gestational) age.
(Rajput *et al.*, 2016)	29			1.40 (0.37-3.69)				*n*=461	Indian	Excluded those with chronic illness, ((family) history of) thyroid illness, consuming thyroid medications, presence of anti-thyroid antibodies or poor obstetrics history	Siemens	≤12th week of gestation, mean 8.58
					1.74 (0.54-4.47)			*n*=469				12-28th week of gestation, mean 19.47
						2.22 (0.70-4.64)		*n*=500				>28th week of gestation, mean 34.36
(Rosario, Carvalho and Calsolari, 2016)	30			0.96 (0.04-2.68)				*n*=660	Brazilian	Excluded those with ((family) history of) thyroid or autoimmune diseases, taking medications, or positive thyroid auto-antibodies	Diagnostic Products Corporation	1st trimester, median 9th week gestation
(Sekhri *et al.*, 2016)	31		2.39 (0.58-4.19)					*n*=124	Indian	Excluded those with thyroid disease or thyroid auto-antibodies	Roche	Mean age 29.8±6.4 years
				1.89 (0.09-6.65)				*n*=86				Mean age 24.5±3.5 years
					2.31 (0.51-6.66)							
						2.26 (0.91-4.86)						
(Springer, Zima and Limanova, 2009)	32			1.28 (0.05-4.80)				*n*= 5520	Czech	Excluded women with thyroid disease and TSH<0.01 or TSH>8	Siemens	9-11th week of gestation
(Sriphrapradang *et al.*, 2014)	33		1.62 (0.36-5.26)					*n*= 1047	Thai	Disease free population, excluded TSH>20	Roche	14+ years old
(Soldin *et al.*, 2010)	NA	(1.12-5.50)						*n*=171	African-American and White American	Total sample	Abbott	<2 months
		(1.12-4.47)						*n*=152				2-11 months
		(1.00-4.37)						*n*=203				12-23 months
		(0.85-4.07)						*n*=264				2-5 years
		(0.89-4.07)						*n*=697				5-10 years
		(0.67-3.72)						*n*= 1063				10-15 years
		(0.47-3.63)						*n*=877				15-20 years
(Stricker *et al.*, 2007)	34			1.04 (0.09-2.83)				*n*=783	Swiss	Excluded those with thyroid auto-immunity, miscarriage or foetal abnormalities	Abbott	<6-12th week
					1.02 (0.20-2.79)			*n*=528				>12-24th week
						1.14 (0.31-2.90)		*n*=501				>24th week until term

Supplementary Table 1: Female TSH reference intervals (2.5^{th} and 97.5^{th} percentiles) published from 2000 according to reproductive life stage

Paper authors	Reference number	Childhood	Adulthood (not pregnant)	Pregnancy 1st trimester	Pregnancy 2nd trimester	Pregnancy 3rd trimester	Post-reproductive	Nr of subjects	Ethnicity	Selection participants	Assay manufacturer	Any comments, including (gestational) age.
(Wang *et al.*, 2009)	35			1.55 (0.41-3.57)				*n*=30	Chinese	Excluded those with (history) of thyroid disease or taking medications affecting thyroid function	Chemiluminescent immune-assay (CLIA), manufacturer not specified	
					1.99 (0.37-8.70)			*n*=47				
						2.41 (0.15-6.96)		*n*=50				
			1.78 (<0.002-5.53)					*n*=49				Lactating (<6 months postpartum)
(Xing *et al.*, 2016)	36		2.69 (0.69-5.78)					*n*=237	Chinese	Excluded those with (history of) thyroid disease or thyroid auto-antibodies, taking medications that affect thyroid function, any other chronic diseases, complicated pregnancy-associated or twin pregnancy	Siemens	19-43 years, mean 29.1
				1.30 (0.07-3.96)				*n*=398				4.3-12.9 gestational weeks, median 12.0
					1.64 (0.27-4.53)			*n*=797				13-27.9 gestational weeks, median 17.6
						2.10 (0.48-5.40)		*n*=1345				28.0-41.9 gestational weeks, median 37.6
(Yan *et al.*, 2011)	37		2.15 (0.50-4.80)					*n*=153	Chinese	Excluded those with thyroid disease or thyroid auto-antibodies	Bayer	18-42 years
				1.50 (0.03-4.51)				*n*=168				5-13th gestational week, mean 9.4
					1.80 (0.05-4.50)			*n*=168				14-27th gestational week, mean 20
						1.81 (0.47-4.54)		*n*=169				28-41st gestational week, mean 35.3

Supplementary Table 1: Female TSH reference intervals (2.5th and 97.5th percentiles) published from 2000 according to reproductive life stage

TABLE 2: RESULTS PUBMED SEARCH TO BE INCLUDED IN FIGURE 2 AND SUPPLEMENTARY FIGURES

Paper authors	Reference Number	Childhood	Adulthood (not pregnant)	Pregnancy trimester 1st	2nd	3rd	Post-reproductive	Nr of subjects	Ethnicity	Selection participants	Assay manufacturer	Any comments, including (gestational) age.
(Aktas *et al.*, 2017)	38	5.6 (1.1-20.4)						*n*=7616	Turkish	Excluded neonates with special conditions requiring more than 4 days of hospitalization, including neonates with serious congenital abnormalities.	Roche	Pre- and term neonates, age measured by gestational age is 34-41 weeks. Blood samples taken at the end of the first week of life.
(Amouzegar *et al.*, 2013)	39		1.65 (0.32-5.63)					*n*=1264	Iranian	Excluded those with known history of thyroid disease, taking thyroid altering medications, pregnant/ breastfeeding women, individuals that were positive for anti-TPO antibodies and those with extremely high or low TSH values.	Roche	20-60+ years
(Andersen *et al.*, 2019)[1]	40			(0.12-2.38)				*n*=1600	Danish	Excluded those without live births, twin pregnancies, those with known thyroid or autoimmune disease, taking thyroid-interfering medication or positive for thyroid autoantibodies.	Siemens	11th gestational week, median maternal age was 29 years (range 16–51 years)
					(0.31-2.93)			*n=123*				15th-20th gestational week, median maternal age was 29 years (range 16–51 years)
(Castillo Lara *et al.*, 2017)	41			2.040 (0.131-4.181)				*n=100*	Spanish	Excluded those with TPO autoantibodies, twin pregnancies, diabetes, hypertension, known thyroid disease or abnormal levels of free T4	Roche	First ten weeks gestation. Mean age of the mothers was 32.13 ± 5.21 years.

Supplementary Table 1: Female TSH reference intervals (2.5^{th} and 97.5^{th} percentiles) published from 2000 according to reproductive life stage

Paper authors	Reference number	Childhood	Adulthood (not pregnant)	Pregnancy 1st trimester	Pregnancy 2nd trimester	Pregnancy 3rd trimester	Post-reproductive	Nr of subjects	Ethnicity	Selection participants	Assay manufacturer	Any comments, including (gestational) age.
(Fan *et al*., 2016)	42			(0.06-3.01)	(0.29-2.93)	(0.47-4.24)		*n*=200	Chinese	Excluded those with pregnancy-related complications, TPO autoantibodies, known thyroid dysfunction, taking medicines influencing thyroid function.	Abbott	T1:9th-13th week gestation; T2:16th-20th week gestation; T3:37th-40th week gestation;
				(0.09-4.13)	(0.44-4.24)	(0.69-5.50)					Roche	
(Feng *et al*., 2014)	43		(0.335–4.859)					*n*=4114	Uygur and Han Chinese	Excluded those taking thyroid medication, with abnormal thyroid hormone levels or thyroid autoantibodies or pregnancy.	Roche	25-45 years
							(0.353-4.447)	*n*=3,936				45-65 years
							(0.247–4.612)	*n*=507				65-85 years
(Flores-Rebollar *et al*., 2015)	44		1.90 (0.69-5.34)					*n*=127	Mexican	Excluded those with thyroid autoantibodies, history of thyroid dysfunction, goiter, drug use (other than estrogen), no thyroid structural abnormalities, nodules, or clinical dysthyroidism.	Izotop	18-40 years
(Gunapalasingham *et al*., 2019)[1]	45	2.2 (0.9-5.3)						*n*=1435 for whole sample, number of participants per age not specified	Danish	Excluded those taking medications known to affect serum concentration of thyroid hormones	Roche	6 years old
		2.3 (1.0-5.4)										11 years old
			2.2 (0.9-5.2)									17 years old
			2.2 (0.9-5.1)									18 years old
(Han *et al*., 2018)	46			1.44 (0.59-3.54)				*n*=188	Chinese	Excluded those with a personal or family history of thyroid disease, goiter, twin pregnancies and complications.	Siemens	1-12 weeks of gestation
					1.78 (0.80-4.46)			*n*=133				13- 28 weeks of gestation
						2.10 (0.72-4.19)		*n*=157				29- 40 weeks of gestation

Supplementary Table 1: Female TSH reference intervals (2.5th and 97.5th percentiles) published from 2000 according to reproductive life stage

Paper authors	Reference number	Childhood	Adulthood (not pregnant)	Pregnancy 1st trimester	Pregnancy 2nd trimester	Pregnancy 3rd trimester	Post-repro-ductive	Nr of subjects	Ethnicity	Selection participants	Assay manu-facturer	Any comments, including (gesta-tional) age.
(Ho *et al*., 2017)[1]	47			0.58 (0.01-2.88)	1.18 (0.33-3.33)	1.16 (0.26-3.48)		*n*=283	Chinese	Excluded those with history of thyroid disease or thyroid medication, multiple pregnancies, chronic medical conditions, pregnancies complicated by fetal anomalies, pregnancies ending in termination,	Abbott	9-14, 18-22, 28-32 weeks of gestation corresponds to 1st, 2nd and 3rd trimester respectively
				0.84 (0.01-2.45)	1.42 (0.35-3.64)	1.32 (0.43-4.06)		*n*=143	Malay			
				0.69 (0.02-2.21)	1.32 (0.26-2.96)	1.14 (0.26-3.03)		*n*=71	Indian			
(Jiang *et al*., 2019)[1]	48			0.62 (0.01-3.28)				*n*=139	Chinese	Excluded those with TPO antibodies, known thyroid disease history or receiving thyroid-related medications	Siemens	7-12 weeks of gestation; twin pregnancies
				1.20 (0.10-3.21)				*n=417*				7-12 weeks of gestation; singleton pregnancies
(Joosen *et al*., 2016)	49			1.30 (0.11-3.39)				*n=97*	Dutch	Excluded those with diseases of pregnancy, premature deliveries, diabetes or other disease, or testing positive for TPO autoantibodies.	Roche	9-13th week of gestation
					1.44 (0.25-3.38)			*n=94*				27-29th week of gestation
						1.83 (0.51-3.85)		*n=94*				36-39th week of gestation
			1.49 (0.53-2.92)					*n=93*				4-13 weeks postpartum
(Kianpour *et al*., 2019)	50			1.50 (0.20-4.60)				*n=185*	Iranian	Excluded those with multiple gestation, goiter, thyroid nodules, autoimmune or other diseases, positive TPO anti-body, known thyroid disease, taking thyroid-altering medications, or inadequate iodine status.	Siemens	9.6th weeks of gestation (median)
			2.10 (0.59-5.60)					*n=256*				15–45 years old

Supplementary Table 1: Female TSH reference intervals (2.5th and 97.5th percentiles) published from 2000 according to reproductive life stage

Paper authors	Reference number	Childhood	Adulthood (not pregnant)	Pregnancy 1st trimester	Pregnancy 2nd trimester	Pregnancy 3rd trimester	Post-reproductive	Nr of subjects	Ethnicity	Selection participants	Assay manufacturer	Any comments, including (gestational) age.
(Langén *et al.*, 2014)	51		1.29 (0.39-3.22)					*n=745*	Finnish	Excluded those taking thyroid hormones or antithyroid agents, with a personal history of thyroid disease or goiter, TPO antibodies, ongoing pregnancy or breast-feeding.	Abbott	30->60 years old
(Laurberg *et al.*, 2016)	52			1.00 (0.37-2.19)				*n=639*	Danish	Excluded those taking thyroid altering medication, with thyroid disease or other autoimmune disease before, during or after the pregnancy, or with abnormal thyroid hormone levels.	Siemens	5-6 weeks of gestation
					1.00 (0.11-2.65)			*n=524*				13-19 weeks of gestation
(Li *et al.*, 2011)[1]	53	2.12 (0.69-6.03)						*n=164*	Chinese	Excluded those with known history of thyroid disease, taking thyroid-altering medicines, pregnancy or breastfeeding.	Diagnostic Products Corporation	14-19 years old
			1.73 (0.35-5.78)					*n=108*				25-29 years old
			1.55 (0.44-5.79)					*n=397*				40-49 years old
							1.53 (0.48-5.34)	*n=75*				60-69 years old
(Lim, 2017)	54	2.43 (0.60-7.39)						*n=934*	Korean	Excluded those with positive TPO antibodies, goiter, personal or family history of thyroid disease, thyroid autoimmune diseases, or smokers.	Roche	10-18 years old
(Loh *et al*, 2015)[1]	55	(0.82-12.08)							Singaporean	Excluded those with extreme TSH or T4 values.	Siemens	0-1 week old
		(0.74-5.68)										2.1 months to 4.0 years
		(0.62-4.52)										7.1-10.0 years
		(0.47-3.74)										12.1-20.0 years

Supplementary Table 1: Female TSH reference intervals (2.5th and 97.5th percentiles) published from 2000 according to reproductive life stage

Paper authors	Reference number	Childhood	Adulthood (not pregnant)	Pregnancy 1st trimester	Pregnancy 2nd trimester	Pregnancy 3rd trimester	Post-reproductive	Nr of subjects	Ethnicity	Selection participants	Assay manufacturer	Any comments, including (gestational) age.
(Margozzini *et al*., 2017)	56			1.82 (0.01-6.33)				*n=720*	Chilean	Excluded those with multiple gestations, prior history of thyroid dysfunction, diabetes, use of thyroid-altering medications, a history of thyroid surgery or radioactive iodine treatment	Roche	<9-14th week of gestation Underweight
				1.90 (0.05-6.08)								<9-14th week of gestation Normal Weight
				1.87 (0.33-5.94)								<9-14th week of gestation Overweight
				2.27 (0.46-6.61)								<9-14th week of gestation Obesity
(Moon *et al*., 2015)	57		1.93 (0.56-4.98)					*n=206*	Korean	Excluded those with history of thyroid disease or goiter; any pregnancy-associated complications, premature birth, or other adverse maternal-fetal outcomes; twin pregnancy; and positive thyroid-specific auto-antibody	Roche	
				1.15 (0.01-4.10)				*n=120*				12.1 (6.3-13.7) weeks of gestation
					1.55 (0.01-4.26)			*n=211*				16.6 (14.4-27.7) weeks of gestation
						2.12 (0.15-4.57)		*n=134*				39.6 (28.1-41.8) weeks of gestation
(Nazarpour *et al*., 2018)	58			1.72 (0.14-6.14)				*n=314*	Iranian	Excluded those with chronic medical disorders, taking thyroid-altering medications, history of blood borne diseases or thyroid disorders, thyroid surgery or radioactive iodine treatment, family history of thyroid disease, history of miscarriages, or foetal abnormalities.	Izotop	<14 weeks of gestation
					1.91 (0.43-4.64)							20-24 weeks of gestation
						1.80 (0.63-3.90)						30-34 weeks of gestation

Supplementary Table 1: Female TSH reference intervals (2.5th and 97.5th percentiles) published from 2000 according to reproductive life stage

Paper authors	Reference number	Childhood	Adulthood (not pregnant)	Pregnancy 1st trimester	Pregnancy 2nd trimester	Pregnancy 3rd trimester	Post-reproductive	Nr of subjects	Ethnicity	Selection participants	Assay manufacturer	Any comments, including (gestational) age.
(Ollero *et al*., 2019)	59			1.33 (0.13-4.16)				*n=288*	Spanish	Excluded those with multiple gestation pregnancies, positive thyroid autoimmunity, abnormally high or low thyroid hormone levels, or clinically significant thyroid nodules.	Abbott	9 weeks of gestation (median)
					1.42 (0.31-3.73)			*n=252*				15 weeks of gestation (median)
						1.94 (0.58-4.36)		*n=236*				36 weeks of gestation (median)
(Su *et al*., 2019)[1]	60		2.00 (0.39-5.13)					*n= 359,895*	Chinese	Excluded those with abnormal thyroid hormone values, thyroid-related diseases or any other diseases that can cause changes in TSH levels, family disease history, or taking any medications	Different labs used ELISA kits with the same reference intervals (manufacturers not specified)	15-55 years old
(Tozzoli *et al*., 2018)[1]	61	2.29 (0.53-4.81)						*n=478*	Italian	Excluded those with detectable thyroid autoantibodies, personal or family history of thyroid dysfunction, visible or palpable goiter, or taking medications	Siemens	0-4 years old
		2.0 (0.76-3.70)						*n=1470*				10-14 years old
			2.0 (0.47-3.91)					*n=3874*				20-24 years old
			1.84 (0.37-3.74)					*n=6626*				30-34 years old
			1.74 (0.40-3.34)					*n-8239*				40-45 years old
							1.76 (0.34-3.50)	*n=7190*				50-54 years old
							1.71 (0.22-3.44)	*n=6534*				60-64 years old
							1.64 (0.22-3.53)	*n=5913*				70-74 years old
							1.54 (0.18-3.54)	*n=4182*				80-84 years old
(Veltri *et al*., 2017)[1]	62			(0.06- 3.74)				*n=1459*	Belgian (mix of women with African or Caucasian background)	Excluded those with twin pregnancies, thyroid autoimmunity, taking thyroid altering medications or positive for TPO antibodies	Siemens	11th week of gestation (median)

Supplementary Table 1: Female TSH reference intervals (2.5th and 97.5th percentiles) published from 2000 according to reproductive life stage

Paper authors	Reference number	Childhood	Adulthood (not pregnant)	Pregnancy 1st trimester	Pregnancy 2nd trimester	Pregnancy 3rd trimester	Post-repro-ductive	Nr of subjects	Ethnicity	Selection participants	Assay manu-facturer	Any comments, including (gesta-tional) age.
(Wang *et al*., 2018)	63		1.778 (1.089-2.709)					*n=8159*	Chinese	Excluded individuals with clear thyroid disorder, but medication history was not available.	Siemens	18-29 years
			1.740(1.059 -2.671)					*n=15019*				30-39 years
			1.822(1.100 -2.823)					*n=13932*				40-49 years
							1.812(1.047 -2.929)	*n=19672*				50-64 years
							1.766(1.003 - 2.984)	*n=5435*				>65 years
(Yang *et al*., 2019)	64			1.17 (0.03-3.64)				*n=46,262*	Chinese	Excluded those with twin pregnancies, thyroid interfering medication, or women who had pre-existing thyroid disease	Abbott	9-13th week of gestation
						1.47 (0.38-3.67)						32-36th week of gestation
(Zhang *et al*., 2016)[1]	65			1.65 (0.35-4.13)				*n=99*	Chinese	Excluded those with multiple pregnancies, a personal or family history of thyroid disease, visible or palpable goiter confirmed by ultrasound, those taking thyroid-altering medication	Roche	8th week of gestation
					1.98 (0.67-3.93)			*n=84*				20th week of gestation
						1.93 (0.66-4.23)		*n=73*				36th week of gestation
			2.02 (0.69-4.96)					*n=78*				12 weeks postpartum

Supplementary Table 1: Female TSH reference intervals (2.5th and 97.5th percentiles) published from 2000 according to reproductive life stage

Bibliography

Abraham, R., Srinivasa Murugan, V., Pukazhvanthen, P. and Sen, S. K. (2009) 'Thyroid disorders in women of Puducherry', *Indian Journal of Clinical Biochemistry*, 24(1), pp. 52–59. doi: 10.1007/s12291-009-0009-y.

Adeniran, K. A., Okolo, A. A. and Onyiriuka, A. N. (2010) 'Thyroid profile of term appropriate for gestational age neonates in Nigeria: A forerunner to screening for congenital hypothyroidism', *Journal of Tropical Pediatrics*, 56(5), pp. 329–332. doi: 10.1093/tropej/fmp143.

Ahmed, Z., Khan, M. A., ul Haq, A., Attaullah, S. and ur Rehman, J. (2009) 'Effect of race, gender and age on thyroid and thyroid stimulating hormone levels in North West Frontier Province, Pakistan.', *Journal of Ayub Medical College, Abbottabad : JAMC*, 21(3), pp. 21–24.

Aktas, O. N., Gursoy, T., Soysal, E., Esencan, E. and Ercin, S. (2017) 'Thyroid hormone levels in late preterm, early term and term infants: A study with healthy neonates revealing reference values and factors affecting thyroid hormones', *Journal of Pediatric Endocrinology and Metabolism*, 30(11), pp. 1191–1196. doi: 10.1515/jpem-2017-0215.

Aldrimer, M., Ridefelt, P., Rödöö, P., Niklasson, F., Gustafsson, J. and Hellberg, D. (2012) 'Reference intervals on the Abbot Architect for serum thyroid hormones, lipids and prolactin in healthy children in a population-based study', *Scandinavian Journal of Clinical and Laboratory Investigation*, 72(4), pp. 326–332. doi: 10.3109/00365513.2012.667571.

Almomin, A., Mansour, A. and Sharief, M. (2016) 'Trimester-Specific Reference Intervals of Thyroid Function Testing in Pregnant Women from Basrah, Iraq Using Electrochemiluminescent Immunoassay', *Diseases*, 4(4), p. 20. doi: 10.3390/diseases4020020.

Amouzegar, A., Delshad, H., Mehran, L., Tohidi, M., Khafaji, F. and Azizi, F. (2013) 'Reference limit of thyrotropin (TSH) and free thyroxine (FT4) in thyroperoxidase positive and negative subjects: A population based study', *Journal of Endocrinological Investigation*, 36(11), pp. 950–954. doi: 10.3275/9033.

Andersen, S. L., Andersen, S., Carlé, A., Christensen, P. A., Handberg, A., Karmisholt, J., Knøsgaard, L., Kristensen, S. R., Bülow Pedersen, I. and Vestergaard, P. (2019) 'Pregnancy Week-Specific Reference Ranges for Thyrotropin and Free Thyroxine in the North Denmark Region Pregnancy Cohort', *Thyroid*, 29(3), pp. 430–438. doi: 10.1089/thy.2018.0628.

Aoki, Y., Belin, R. M., Clickner, R., Jeffries, R., Phillips, L. and Mahaffey, K. R. (2007) 'Serum TSH and Total T4 in the United States Population and Their Association With Participant Characteristics: National Health and Nutrition Examination Survey (NHANES 1999–2002)', *Thyroid*, 17(12), pp. 1211–1223. doi: 10.1089/thy.2006.0235.

Ashoor, G., Kametas, N. A., Akolekar, R., Guisado, J. and Nicolaides, K. H. (2010) 'Maternal Thyroid Function at 11–13 Weeks of Gestation Ghalia', *Fetal Diagnosis and Therapy*, 27, pp. 156–163.

Bailey, D., Colantonio, D., Kyriakopoulou, L., Cohen, A. H., Chan, M. K., Armbruster, D. and Adeli, K. (2013) 'Marked biological variance in endocrine and biochemical markers in childhood: Establishment of pediatric reference intervals using healthy community children from the CALIPER cohort', *Clinical Chemistry*, 59(9), pp. 1393–1403. doi: 10.1373/clinchem.2013.204222.

Bliddal, S., Boas, M., Hilsted, L., Friis-Hansen, L., Tabor, A. and Feldt-Rasmussen, U. (2015) 'Thyroid function and autoimmunity in Danish pregnant women after an iodine fortification program and associations with obstetric outcomes', *European Journal of Endocrinology*, 173(6), pp. 709–718. doi: 10.1530/EJE-15-0358.

Bliddal, S., Feldt-Rasmussen, U., Boas, M., Faber, J., Juul, A., Larsen, T. and Precht, D. H. (2014) 'Gestational age-specific reference ranges from different laboratories misclassify pregnant women's thyroid status: Comparison of two longitudinal prospective cohort studies', *European Journal of Endocrinology*, 170(2), pp. 329–339. doi: 10.1530/EJE-13-0672.

Boucai, L., Hollowell, J. G. and Surks, M. I. (2011) 'An approach for development of age-, gender-, and ethnicity-specific thyrotropin reference limits', *Thyroid*, 21(1), pp. 5–11. doi: 10.1089/thy.2010.0092.

Boucai, L. and Surks, M. I. (2009) 'Reference limits of serum TSH and free T4 are significantly influenced by race and age in an urban outpatient medical practice', *Clinical Endocrinology*, 70(5), pp. 788–793. doi: 10.1111/j.1365-2265.2008.03390.x.

Supplementary Table 1: Female TSH reference intervals (2.5th and 97.5th percentiles) published from 2000 according to reproductive life stage

Cai, J., Fang, Y., Jing, D., Xu, S., Ming, J., Gao, B., Shen, H., Zhang, R. and Ji, Q. (2016) 'Reference intervals of thyroid hormones in a previously iodine-deficient but presently more than adequate area of Western China: A population-based survey', *Endocrine Journal*, 63(4), pp. 381–388. doi: 10.1507/endocrj.EJ15-0574.

Castillo Lara, M., Vilar Sánchez, Á., Cañavate Solano, C., Soto Pazos, E., Iglesias Álvarez, M., González Macías, C., Ayala Ortega, C., Moreno Corral, L. J. and Fernández Alba, J. J. (2017) '"Hypothyroidism screening during first trimester of pregnancy"', *BMC Pregnancy and Childbirth*. BMC Pregnancy and Childbirth, 17(1), pp. 1–7. doi: 10.1186/s12884-017-1624-x.

Chaler, E. A., Fiorenzano, R., Chilelli, C., Llinares, V., Areny, G., Herzovich, V., Maceiras, M., Lazzati, J. M., Mendioroz, M., Rivarola, M. A. and Belgorosky, A. (2012) 'Age-specific thyroid hormone and thyrotropin reference intervals for a pediatric and adolescent population', *Clinical Chemistry and Laboratory Medicine*, 50(5), pp. 885–890. doi: 10.1515/cclm-2011-0495.

Chan, A. O. K., Iu, Y. P. and Shek, C. C. (2011) 'The reference interval of thyroid-stimulating hormone in Hong Kong Chinese', *Journal of Clinical Pathology*, 64(5), pp. 433–436. doi: 10.1136/jcp.2010.087627.

Clerico, A., Trenti, T., Aloe, R., Dittadi, R., Rizzardi, S., Migliardi, M., Musa, R., Dipalo, M., Prontera, C., Masotti, S., Musetti, V., Tozzoli, R., Padoan, A. and Bagnasco, M. (2019) 'A multicenter study for the evaluation of the reference interval for TSH in Italy (ELAS TSH Italian Study)', *Clinical Chemistry and Laboratory Medicine*, 57(2), pp. 259–267. doi: 10.1515/cclm-2018-0541.

Dashe, J. S., Casey, B., Wells, E., Mcintire, D., Byrd, W. E., Leveno, K. and Cunningham, G. F. (2005) 'Thyroid-stimulating hormone in singleton and twin pregnancy: Importance of gestational age-specific reference ranges', *Obstetrics and Gynecology*, 106(4), pp. 753–757. doi: 10.1097/01.AOG.0000195268.33533.8a.

Dhatt, G. S., Griffin, G. and Agarwal, M. M. (2006) 'Thyroid hormone reference intervals in an ambulatory Arab population on the Abbott Architect i2000 immunoassay analyzer', *Clinica Chimica Acta*, 364(1–2), pp. 226–229. doi: 10.1016/j.cccn.2005.07.003.

Dhatt, G. S., Jayasundaram, R., Wareth, L. A., Nagelkerke, N., Jayasundaram, K., Darwish, E. A. and Lewis, A. (2006) 'Thyrotrophin and free thyroxine trimester-specific reference intervals in a mixed ethnic pregnant population in the United Arab Emirates', *Clinica Chimica Acta*, 370(1–2), pp. 147–151. doi: 10.1016/j.cca.2006.02.008.

Donovan, L. E., Metcalfe, A., Chin, A., Yamamoto, J. M., Virtanen, H., Johnson, J. A. and Krause, R. (2019) 'A Practical Approach for the Verification and Determination of Site- and Trimester-Specific Reference Intervals for Thyroid Function Tests in Pregnancy', *Thyroid*, 29(3), pp. 412–419. doi: 10.1089/thy.2018.0439.

Ehrenkranz, J., Bach, P. R., Snow, G. L., Schneider, A., Lee, J. L., Ilstrup, S., Bennett, S. T. and Benvenga, S. (2015) 'Circadian and Circannual Rhythms in Thyroid Hormones: Determining the TSH and Free T4 Reference Intervals Based Upon Time of Day, Age, and Sex', *Thyroid*, 25(8), pp. 954–961. doi: 10.1089/thy.2014.0589.

Elhaj, E. T., Adam, I., Ahmed, M. A. and Lutfi, M. F. (2016) 'Trimester-specific thyroid hormone reference ranges in Sudanese women Biomedical research', *BMC Physiology*. BMC Physiology, 16(1), pp. 1–8. doi: 10.1186/s12899-016-0025-0.

Eltom, A., Eltom, M., Elnagar, B., Elbagir, M. and Gebre-Medhin, M. (2000) 'Changes in iodine metabolism during late pregnancy and lactation: A longitudinal study among Sudanese women', *European Journal of Clinical Nutrition*, 54(5), pp. 429–433. doi: 10.1038/sj.ejcn.1600992.

Fan, J. X., Yang, S., Qian, W., Shi, F. T. and Huang, H. F. (2016) 'Comparison of the reference intervals used for the evaluation of maternal thyroid function during pregnancy using sequential and nonsequential methods', *Chinese Medical Journal*, 129(7), pp. 785–791. doi: 10.4103/0366-6999.178954.

Feng, Y., Bian, W., Mu, C., Xu, Y., Wang, F., Qiao, W. and Huang, Y. (2014) 'Establish and verify TSH reference intervals using optimized statistical method by analyzing laboratory-stored data', *Journal of Endocrinological Investigation*, 37(3), pp. 277–284. doi: 10.1007/s40618-013-0031-z.

Flores-Rebollar, A., Moreno-Castañeda, L., Vega-Servín, N. S., López-Carrasco, G. and Ruiz-Juvera, A. (2015) 'Determination of thyrotropin reference values in an adult Mexican population', *Endocrinologia y Nutricion*, 62(2), pp. 56–63. doi: 10.1016/j.endonu.2014.10.003.

Supplementary Table 1: Female TSH reference intervals (2.5th and 97.5th percentiles) published from 2000 according to reproductive life stage

Fontes, R., Coeli, C. R., Aguiar, F. and Vaisman, M. (2013) 'Reference interval of thyroid stimulating hormone and free thyroxine in a reference population over 60 years old and in very old subjects (over 80 years): Comparison to young subjects', *Thyroid Research*, 6(1), pp. 2–9. doi: 10.1186/1756-6614-6-13.

Gilbert, R. M., Hadlow, N. C., Walsh, J. P., Fletcher, S. J., Brown, S. J., Stuckey, B. G. and Lim, E. M. (2008) 'Assessment of thyroid function during pregnancy: first-trimester (weeks 9–13) reference intervals derived from Western Australian women', *MJA*, 189(5), pp. 7–10.

Guan, H., Shan, Z., Teng, X., Li, Y., Teng, D., Jin, Y., Yu, X., Fan, C., Chong, W., Yang, F., Dai, H., Yu, Y., Li, J., Chen, Y., Zhao, D., Shi, X., Hu, F., Mao, J., Gu, X., *et al*. (2008) 'Influence of iodine on the reference interval of TSH and the optimal interval of TSH: Results of a follow-up study in areas with different iodine intakes', *Clinical Endocrinology*, 69(1), pp. 136–141. doi: 10.1111/j.1365-2265.2007.03150.x.

Gunapalasingham, G., Frithioff-Bøjsøe, C., Lund, M. A. V., Hedley, P. L., Fonvig, C. E., Dahl, M., Pedersen, O., Christiansen, M., Hansen, T., Lausten-Thomsen, U. and Holm, J. C. (2019) 'Reference values for fasting serum concentrations of thyroid-stimulating hormone and thyroid hormones in healthy Danish/North-European white children and adolescents', *Scandinavian Journal of Clinical and Laboratory Investigation*. Taylor & Francis, 79(1–2), pp. 129–135. doi: 10.1080/00365513.2019.1581945.

Haddow, J. E., Knight, G. J., Palomaki, G. E., McClain, M. R. and Pulkkinen, A. J. (2004) 'The reference range and within-person variability of thyroid stimulating hormone during the first and second trimesters of pregnancy', *Journal of Medical Screening*, 11(4), pp. 170–174. doi: 10.1258/0969141042467340.

Hadlow, N. C., Rothacker, K. M., Wardrop, R., Brown, S. J., Lim, E. M. and Walsh, J. P. (2013) 'The relationship between TSH and free T4in a large population is complex and nonlinear and differs by age and sex', *Journal of Clinical Endocrinology and Metabolism*, 98(7), pp. 2936–2943. doi: 10.1210/jc.2012-4223.

Han, L., Zheng, W., Zhai, Y., Xie, X., Zhang, J., Zhang, S., Zhao, Z. and Cao, Z. (2018) 'Reference intervals of trimester-specific thyroid stimulating hormone and free thyroxine in Chinese women established by experimental and statistical methods', *Journal of Clinical Laboratory Analysis*, 32(4), pp. 4–9. doi: 10.1002/jcla.22344.

Hickman, P. E., Koerbin, G., Simpson, A., Potter, J. M., Hughes, D. G., Abhayaratna, W. P., West, N., Glasgow, N., Armbruster, D., Cavanaugh, J. and Reed, M. (2017) 'Using a thyroid disease-free population to define the reference interval for TSH and free T4 on the Abbott Architect analyser', *Clinical Endocrinology*, 86(1), pp. 108–112. doi: 10.1111/cen.13143.

Ho, C. K. M., Tan, E. T. H., Ng, M. J., Yeo, G. S. H., Chern, B., Tee, N. W. S., Kwek, K. Y. C. and Tan, K. H. (2017) 'Gestational age-specific reference intervals for serum thyroid hormone levels in a multi-ethnic population', *Clinical Chemistry and Laboratory Medicine*, 55(11), pp. 1777–1788. doi: 10.1515/cclm-2016-0790.

Hoermann, R., Larisch, R., Dietrich, J. W. and Midgley, J. E. M. (2016) 'Derivation of a multivariate reference range for pituitary thyrotropin and thyroid hormones: Diagnostic efficiency compared with conventional single-reference method', *European Journal of Endocrinology*, 174(6), pp. 735–743. doi: 10.1530/EJE-16-0031.

Hollowell, J. G., Staehling, N. W., Dana Flanders, W., Harry Hannon, W., Gunter, E. W., Spencer, C. A. and Braverman, L. E. (2002) 'Serum TSH, T4, and thyroid antibodies in the United States population (1988 to 1994): National Health and Nutrition Examination Survey (NHANES III)', *Journal of Clinical Endocrinology and Metabolism*, 87(2), pp. 489–499. doi: 10.1210/jcem.87.2.8182.

Inal, T. C., Serteser, M., Coskun, A., Özpinar, A. and Ünsal, I. (2010) 'Indirect reference intervals estimated from hospitalized population for thyrotropin and free thyroxine', *Croatian Medical Journal*, 51(2), pp. 124–130. doi: 10.3325/cmj.2010.51.124.

Ishaku, A. A., Shabbal, D. M. and Isichei, C. (2017) 'Reference interval of thyroxine and thyrotropin of healthyterm Nigerian newborns in Jos University of Teaching Hospital', *Jos Journal of Medicine*, 11(1), pp. 26-30–30.

Ittermann, T., Khattak, R. M., Nauck, M., Cordova, C. M. M. and Völzke, H. (2015) 'Shift of the TSH reference range with improved iodine supply in Northeast Germany', *European Journal of Endocrinology*, 172(3), pp. 261–267. doi: 10.1530/EJE-14-0898.

Jang, Y. Y., Kim, C. Y., Hwang, T. Y., Kim, K. D. and Lee, C. H. (2008) 'Reference interval of serum thyroid hormones in healthy Korean adults', *Journal of Preventive Medicine and Public Health*, 41(2), pp. 128–134. doi: 10.3961/jpmph.2008.41.2.128.

Supplementary Table 1: Female TSH reference intervals (2.5th and 97.5th percentiles) published from 2000 according to reproductive life stage

Jayasuriya, M. S., Choy, K. W., Chin, L. K., Doery, J., Stewart, A., Bergman, P. and Lu, Z. X. (2018) ‘Reference intervals for neonatal thyroid function tests in the first 7 days of life’, *Journal of Pediatric Endocrinology and Metabolism*, 31(10), pp. 1113–1116. doi: 10.1515/jpem-2018-0007.

Jebasingh, F. K., Salam, R., Meetei, T. L., Singh, P. T., Singh, N. N. and Prasad, L. (2016) ‘Reference intervals in evaluation of maternal thyroid function of Manipuri women’, *Indian Journal of Endocrinology and Metabolism*. Medknow Publications, 20(2), pp. 167–170. doi: 10.4103/2230-8210.176354.

Jeon, M. J., Kim, W. G., Kwon, H., Kim, M., Park, S., Oh, H. S., Han, M., Kim, T. Y., Shong, Y. K. and Kim, W. B. (2017) ‘Excessive Iodine Intake and Thyrotropin Reference Interval: Data from the Korean National Health and Nutrition Examination Survey’, *Thyroid*, 27(7), pp. 967–972. doi: 10.1089/thy.2017.0078.

Jiang, Y. X., Sun, W. J., Zhang, Y., Huang, Y., Huang, Y. Y., Lu, G. Z., Zhang, J. Q., Gao, Y., Yang, H. X. and Guo, X. H. (2019) ‘Thyroid function of twin-pregnant women in early pregnancy’, *Chinese Medical Journal*, 132(17), pp. 2033–2038. doi: 10.1097/CM9.0000000000000381.

Joosen, A. M. C. P., Van Der Linden, I. J. M., De Jong-Aarts, N., Hermus, M. A. A., Ermens, A. A. M. and De Groot, M. J. M. (2016) ‘TSH and fT4 during pregnancy: An observational study and a review of the literature’, *Clinical Chemistry and Laboratory Medicine*, 54(7), pp. 1239–1246. doi: 10.1515/cclm-2015-0629.

Kahapola-Arachchige, K. M., Hadlow, N., Wardrop, R., Lim, E. M. and Walsh, J. P. (2012) ‘Age-specific TSH reference ranges have minimal impact on the diagnosis of thyroid dysfunction’, *Clinical Endocrinology*, 77(5), pp. 773–779. doi: 10.1111/j.1365-2265.2012.04463.x.

Kaluarachchi, D. C., Allen, D. B., Eickhoff, J. C., Dawe, S. J. and Baker, M. W. (2019) ‘Thyroid-stimulating hormone reference ranges for preterm infants’, *Pediatrics*, 144(2). doi: 10.1542/peds.2019-0290.

Kang, M. J., Hwang, I. T. and Chung, H. R. (2018) ‘Excessive iodine intake and subclinical hypothyroidism in children and adolescents aged 6-19 years: Results of the sixth Korean national health and nutrition examination survey, 2013-2015’, *Thyroid*, 28(6), pp. 773–779. doi: 10.1089/thy.2017.0507.

Kapelari, K., Kirchlechner, C., Högler, W., Schweitzer, K., Virgolini, I. and Moncayo, R. (2008) ‘Pediatric reference intervals for thyroid hormone levels from birth to adulthood: A retrospective study’, *BMC Endocrine Disorders*, 8, pp. 1–10. doi: 10.1186/1472-6823-8-15.

Kawahara, K. and Yokoya, S. (2002) ‘Establishment of reference intervals of thyrotropin and free thyroid hormones during the first week of life’, *Clinical Pediatric Endocrinology*, 11(1), pp. 1–9. doi: 10.1297/cpe.11.1.

Kianpour, M., Aminorroaya, A., Amini, M., Feizi, A., Janghorbani, M., Shokri, S., Yamini, S. A., Farghadani, M., Hekmatnia, A. and Gharib, H. (2019) ‘Reference Intervals for Thyroid Hormones during the First Trimester of Gestation: A Report from an Area with a Sufficient Iodine Level’, *Hormone and Metabolic Research*, 51(3), pp. 165–171. doi: 10.1055/a-0855-7128.

Kim, H. J., Cho, Y. Y., Kim, S. W., Kim, T. H., Jang, H. W., Lee, S. Y., Choi, S. J., Roh, C. R., Kim, J. H., Chung, J. H. and Oh, S. Y. (2018) ‘Reference intervals of thyroid hormones during pregnancy in korea, an iodine-replete area’, *Korean Journal of Internal Medicine*, 33(3), pp. 552–560. doi: 10.3904/kjim.2016.051.

Knudsen, N., Bulow, I., Jorgensen, T., Laurberg, P., Ovesen, L. and Perrild, H. (2000) ‘Comparative study of thyroid function and types of thyroid dysfunction in two areas in Denmark with slightly different iodine status’, *European Journal of Endocrinology*, 143(4), pp. 485–491. doi: 10.1530/eje.0.1430485.

Kratzsch, J., Fiedler, G. M., Leichtle, A., Brügel, M., Buchbinder, S., Otto, L., Sabri, O., Matthes, G. and Thiery, J. (2005) ‘New reference intervals for thyrotropin and thyroid hormones based on national academy of clinical biochemistry criteria and regular ultrasonography of the thyroid’, *Clinical Chemistry*, 51(8), pp. 1480–1486. doi: 10.1373/clinchem.2004.047399.

Kratzsch, J., Schubert, G., Pulzer, F., Pfaeffle, R., Koerner, A., Dietz, A., Rauh, M., Kiess, W. and Thiery, J. (2008) ‘Reference intervals for TSH and thyroid hormones are mainly affected by age, body mass index and number of blood leucocytes, but hardly by gender and thyroid autoantibodies during the first decades of life’, *Clinical Biochemistry*, 41(13), pp. 1091–1098. doi: 10.1016/j.clinbiochem.2008.04.007.

Kurioka, H., Takahashi, K. and Miyazaki, K. (2005) ‘Maternal thyroid function during pregnancy and puerperal period’, *Endocrine Journal*, 52(5), pp. 587–591. doi: 10.1507/endocrj.52.587.

Kutluturk, F., Yildirim, B., Ozturk, B., Ozyurt, H., Bekar, U., Sahin, S., Akturk, Y., Akbas, A., Cetin, I. and Etikan, I. (2014) ‘The reference intervals of thyroid

stimulating hormone in healthy individuals with normal levels of serum free thyroxine and without sonographic pathologies', *Endocrine Research*, 39(2), pp. 57–61. doi: 10.3109/07435800.2013.824896.

Kwon, H., Kim, W. G., Jeon, M. J., Han, M., Kim, M., Park, S., Kim, T. Y., Shong, Y. K. and Kim, W. B. (2017) 'Age-specific reference interval of serum TSH levels is high in adolescence in an iodine excess area: Korea national health and nutrition examination survey data', *Endocrine*. Springer US, 57(3), pp. 445–454. doi: 10.1007/s12020-017-1375-5.

La'ulu, S. L. and Roberts, W. L. (2011) 'Ethnic differences in first-trimester thyroid reference intervals', *Clinical Chemistry*, 57(6), pp. 913–915. doi: 10.1373/clinchem.2010.161240.

Lambert-Messerlian, G., McClain, M., Haddow, J. E., Palomaki, G. E., Canick, J. A., Cleary-Goldman, J., Malone, F. D., Porter, T. F., Nyberg, D. A., Bernstein, P., D'Alton, M. E., The FaSTER Research Consortium and OBJECTIVE: (2008) 'First- and second-trimester thyroid hormone reference data in pregnant women: a FaSTER (First- and Second-Trimester Evaluation of Risk for aneuploidy) Research Consortium study', *American Hournal of Obstetrics and Gynaecology*, 62, pp. e1–e6.

Langén, V. L., Niiranen, T. J., Mäki, J., Sundvall, J. and Jula, A. M. (2014) 'Thyroid-stimulating hormone reference range and factors affecting it in a nationwide random sample', *Clinical Chemistry and Laboratory Medicine*, 52(12), pp. 1807–1813. doi: 10.1515/cclm-2014-0287.

Langén, V. L., Niiranen, T. J., Puukka, P., Sundvall, J. and Jula, A. M. (2017) 'Association of thyroid-stimulating hormone with lipid concentrations: an 11-year longitudinal study', *Clinical Endocrinology*, 86(1), pp. 120–127. doi: 10.1111/cen.13151.

Laurberg, P., Andersen, S. L., Hindersson, P., Nohr, E. A. and Olsen, J. (2016) 'Dynamics and predictors of serum tsh and ft4 reference limits in early pregnancy: A study within the danish national birth cohort', *Journal of Clinical Endocrinology and Metabolism*, 101(6), pp. 2484–2492. doi: 10.1210/jc.2016-1387.

Lee, Y. K., Shin, D. Y., Shin, H. and Lee, E. J. (2018) 'Sex-specific genetic influence on thyroidstimulating hormone and free thyroxine levels, and interactions between measurements: KNHANES 2013 2015', *PLoS ONE*, 13(11), pp. 1–12. doi: 10.1371/journal.pone.0207446.

Li, C., Guan, H., Teng, X., Lai, Y., Chen, Y., Yu, J., Li, N., Wang, B., Jiang, F., Wang, J., Fan, C., Wang, H., Zhang, H., Teng, W. and Shan, Z. (2011) 'An epidemiological study of the serum thyrotropin reference range and factors that influence serum thyrotropin levels in iodine sufficient areas of China', *Endocrine Journal*, 58(11), pp. 995–1002. doi: 10.1507/endocrj.K11E-101.

Lim, H. H. (2017) 'Korean pediatric reference intervals for FT4, TSH, and TPO Ab and the prevalence of thyroid dysfunction: A population-based study', *Clinical Biochemistry*. Elsevier, 50(18), pp. 1256–1259. doi: 10.1016/j.clinbiochem.2017.09.024.

Liu, J., Yu, X., Xia, M., Cai, H., Cheng, G., Wu, L., Li, Q., Zhang, Y., Sheng, M., Liu, Y. and Qin, X. (2017) 'Development of gestation-specific reference intervals for thyroid hormones in normal pregnant Northeast Chinese women: What is the rational division of gestation stages for establishing reference intervals for pregnancy women?', *Clinical Biochemistry*. The Canadian Society of Clinical Chemists, 50(6), pp. 309–317. doi: 10.1016/j.clinbiochem.2016.11.036.

Loh, T. P., Sethi, S. K. and Metz, M. P. (2015) 'Paediatric reference interval and biological variation trends of thyrotropin (TSH) and free thyroxine (T4) in an Asian population', *Journal of Clinical Pathology*, 68(8), pp. 642–647. doi: 10.1136/jclinpath-2015-202916.

Lombardo Grifol, M., Gutiérrez Menéndez, M. L., García Menéndez, L. and Valdazo Revenga, M. V. (2013) 'Reference values and variability study of thyroid hormones in pregnant women from El Bierzo', *Endocrinología y Nutrición (English Edition)*. SEEN, 60(10), pp. 549–554. doi: 10.1016/j.endoen.2013.12.008.

Maji, R., Nath, S., Lahiri, S., Saha Das, M., Bhattacharyya, A. R. and Das, H. N. (2014) 'Establishment of trimester-specific reference intervals of serum TSH & fT4 in a pregnant Indian population at North Kolkata', *Indian Journal of Clinical Biochemistry*, 29(2), pp. 167–173. doi: 10.1007/s12291-013-0332-1.

Mankar, J., Sahasrabuddhe, A. and Pitale, S. (2016) 'Trimester specific ranges for thyroid hormones in normal pregnancy', *Thyroid Research and Practice*, 13(3), p. 106. doi: 10.4103/0973-0354.180192.

Männistö, T., Surcel, H. M., Ruokonen, A., Vääräsmäki, M., Pouta, A., Bloigu, A., Järvelin, M. R., Hartikainen, A. L. and Suvanto, E. (2011) 'Early pregnancy reference intervals of thyroid hormone concentrations in a thyroid antibody-negative pregnant population', *Thyroid*, 21(3), pp. 291–298. doi: 10.1089/thy.2010.0337.

Supplementary Table 1: Female TSH reference intervals (2.5th and 97.5th percentiles) published from 2000 according to reproductive life stage

Mansourian, A. R., Ahmadi, A., Mansourian, H. R., Saifi, A., Marjani, A., Veghari, G. R. and Ghaemi, E. (2010) 'Maternal Thyroid Stimulating Hormone Levels During the First Trimester Of Pregnancy At The South-East Of The Caspian Sea In Iran', (3), pp. 2472–2477.

Margozzini, P., Lyng, T., Carvajal, J. and Campusano, C. (2017) 'Early pregnancy thyroid hormone reference ranges in Chilean women : The influence of body mass index', 85(6), pp. 942–948. doi: 10.1111/cen.13127.Early.

Marwaha, R. K., Chopra, S., Gopalakrishnan, S., Sharma, B., Kanwar, R. S., Sastry, A. and Singh, S. (2008) 'Establishment of reference range for thyroid hormones in normal pregnant Indian women', *BJOG: An International Journal of Obstetrics and Gynaecology*, 115(5), pp. 602–606. doi: 10.1111/j.1471-0528.2008.01673.x.

Marwaha, R. K., Tandon, N., Desai, A. K., Kanwar, R., Aggarwal, R., Sastry, A., Singh, S., Narang, A., Ganguly, S. K. and Mani, K. (2010) 'Reference range of thyroid hormones in healthy school-age children: Country-wide data from India', *Clinical Biochemistry*. Elsevier B.V., 43(1–2), pp. 51–56. doi: 10.1016/j.clinbiochem.2009.03.001.

Marwaha, R. K., Tandon, N., Ganie, M. A., Mehan, N., Sastry, A., Garg, M. K., Bhadra, K. and Singh, S. (2013) 'Reference range of thyroid function (FT3, FT4 and TSH) among Indian adults', *Clinical Biochemistry*. Elsevier B.V., 46(4–5), pp. 341–345. doi: 10.1016/j.clinbiochem.2012.09.021.

Mirjanic-Azaric, B., Avram, S., Stojakovic-Jelisavac, T., Stojanovic, D., Petkovic, M., Bogavac-Stanojevic, N., Ignjatovic, S. and Stojanov, M. (2017) 'Direct Estimation of Reference Intervals for Thyroid Parameters in the Republic of Srpska', *Journal of Medical Biochemistry*, 36(2), pp. 137–144. doi: 10.1515/jomb-2017-0008.

Moncayo, H., Dapunt, O. and Moncayo, R. (2007) 'Diagnostic accuracy of basal TSH determinations based on the intravenous TRH stimulation test: An evaluation of 2570 tests and comparison with the literature', *BMC Endocrine Disorders*, 7, pp. 1–5. doi: 10.1186/1472-6823-7-5.

Moon, H. W., Chung, H. J., Park, C. M., Hur, M. and Yun, Y. M. (2015) 'Establishment of trimester-specific reference intervals for thyroid hormones in Korean pregnant women', *Annals of Laboratory Medicine*, 35(2), pp. 198–204. doi: 10.3343/alm.2015.35.2.198.

De Moura Souza, A. and Sichieri, R. (2011) 'Association between serum TSH concentration within the normal range and adiposity', *European Journal of Endocrinology*, 165(1), pp. 11–15. doi: 10.1530/EJE-11-0261.

Murillo-Llorente, M., Fajardo-Montañana, C., Pérez-Bermejo, M., Vila-Candel, R., Gómez-Vela, J. and Velasco, I. (2017) 'Intra-individual variability in TSH levels of healthy women during the first half of pregnancy', *Endocrinología, Diabetes y Nutrición (English ed.)*, 64(6), pp. 288–294. doi: 10.1016/j.endien.2017.08.007.

Musa, I. R., Ali, N. I., Elseed, S. A., Osman, O. E. and Adam, I. (2018) 'Reference intervals of thyroid hormones in Khartoum, Sudan', *BMC Research Notes*. BioMed Central, 11(1), pp. 1–5. doi: 10.1186/s13104-018-3840-5.

Mutlu, M., Karagüzel, G., Aliyazicioğlu, Y., Eyüpoğlu, I., Ökten, A. and Aslan, Y. (2012) 'Reference intervals for thyrotropin and thyroid hormones and ultrasonographic thyroid volume during the neonatal period', *Journal of Maternal-Fetal and Neonatal Medicine*, 25(2), pp. 120–124. doi: 10.3109/14767058.2011.561894.

Nazarpour, S., Ramezani Tehrani, F., Simbar, M., Minooee, S., Rahmati, M., Mansournia, M. A. and Azizi, F. (2018) 'Establishment of trimester-specific reference range for thyroid hormones during pregnancy', *Clinical Biochemistry*. Elsevier, 53(January), pp. 49–54. doi: 10.1016/j.clinbiochem.2018.01.006.

Nazeri, P., Shariat, M., Mehrabi, Y., Mirmiran, P. and Azizi, F. (2018) 'Is there an association between thyrotropin levels within the normal range and birth growth parameters in full-term newborns?', *Journal of Pediatric Endocrinology and Metabolism*, 31(9), pp. 1001–1007. doi: 10.1515/jpem-2017-0519.

O'Leary, P. C., Feddema, P. H., Michelangeli, V. P., Leedman, P. J., Chew, G. T., Knuiman, M., Kaye, J. and Walsh, J. P. (2006) 'Investigations of thyroid hormones and antibodies based on a community health survey: The Busselton thyroid study', *Clinical Endocrinology*, 64(1), pp. 97–104. doi: 10.1111/j.1365-2265.2005.02424.x.

Ollero, M. D., Toni, M., Pineda, J. J., Martínez, J. P., Espada, M. and Anda, E. (2019) 'Thyroid Function Reference Values in Healthy Iodine-Sufficient Pregnant Women and Influence of Thyroid Nodules on Thyrotropin and Free Thyroxine Values', *Thyroid*, 29(3), pp. 421–429. doi: 10.1089/thy.2018.0324.

Supplementary Table 1: Female TSH reference intervals (2.5th and 97.5th percentiles) published from 2000 according to reproductive life stage

Omuse, G., Kassim, A., Kiigu, F., Hussain, S. R. ana and Limbe, M. (2016) 'Reference intervals for thyroid stimulating hormone and free thyroxine derived from neonates undergoing routine screening for congenital hypothyroidism at a university teaching hospital in Nairobi, Kenya: A cross sectional study', *BMC Endocrine Disorders*. BMC Endocrine Disorders, 16(1), pp. 1–8. doi: 10.1186/s12902-016-0107-9.

Panesar, N. S., Li, C. Y. and Rogers, M. S. (2001) 'Reference intervals for thyroid hormones in pregnant Chinese women', *Annals of Clinical Biochemistry*, 38(4), pp. 329–332. doi: 10.1258/0004563011900830.

Park, S. Y., Kim, H. I., Oh, H. K., Kim, T. H., Jang, H. W., Chung, J. H., Shin, M. H. and Kim, S. W. (2018) 'Age- and gender-specific reference intervals of TSH and free T4 in an iodine-replete area: Data from Korean National Health and Nutrition Examination Survey IV (2013–2015)', *PLoS ONE*, 13(2), pp. 1–12. doi: 10.1371/journal.pone.0190738.

Price, A., Obel, O., Cresswell, J., Catch, I., Rutter, S., Barik, S., Heller, S. R. and Weetman, A. P. (2001) 'Comparison of thyroid function in pregnant and non-pregnant Asian and western Caucasian women', *Clinica Chimica Acta*, 308(1–2), pp. 91–98. doi: 10.1016/S0009-8981(01)00470-3.

Rajput, R., Singh, B., Goel, V., Verma, A., Seth, S. and Nanda, S. (2016) 'Trimester-specific reference interval for thyroid hormones during pregnancy at a Tertiary Care Hospital in Haryana, India', *Indian Journal of Endocrinology and Metabolism*. Medknow Publications, 20(6), pp. 810–815. doi: 10.4103/2230-8210.192903.

Raverot, V., Bournaud, C., Sassolas, G., Orgiazzi, J., Claustrat, F., Gaucherand, P., Mellier, G., Claustrat, B., Borson-Chazot, F. and Zimmermann, M. (2012) 'Pregnant French women living in the Lyon area are iodine deficient and have elevated serum thyroglobulin concentrations', *Thyroid*, 22(5), pp. 522–528. doi: 10.1089/thy.2011.0184.

Roelfsema, F. and Veldhuis, J. D. (2013) 'Thyrotropin secretion patterns in health and disease', *Endocrine Reviews*, 34(5), pp. 619–657. doi: 10.1210/er.2012-1076.

Rosario, P. W. and Calsolari, M. R. (2014) 'TSH reference range in older adults: a Brazilian study', *Arquivos Brasileiros de Endocrinologia e Metabologia*, 58(4), pp. 389–393. doi: 10.1590/0004-2730000003065.

Rosario, P. W., Carvalho, M. and Calsolari, M. R. (2016) 'TSH reference values in the first trimester of gestation and correlation between maternal TSH and obstetric and neonatal outcomes: A prospective Brazilian study', *Archives of Endocrinology and Metabolism*, 60(4), pp. 314–318. doi: 10.1590/2359-3997000000132.

Rosario, P. W., Cezar, A., Xavier, M. and Calsolari, M. R. (2010) 'TSH reference values for adult', *Arq Bras Endocrinol Metab*, 54(7), pp. 603–6.

Ross, H. A., Den Heijer, M., Hermus, A. R. M. M. and Sweep, F. C. G. J. (2009) 'Composite reference interval for thyroid-stimulating hormone and free thyroxine, comparison with common cutoff values, and reconsideration of subclinical thyroid disease', *Clinical Chemistry*, 55(11), pp. 2019–2025. doi: 10.1373/clinchem.2009.124560.

Schalin-Jäntti, C., Tanner, P., Välimäki, M. J. and Hämäläinen, E. (2011) 'Serum TSH reference interval in healthy Finnish adults using the Abbott Architect 2000i Analyzer', *Scandinavian Journal of Clinical and Laboratory Investigation*, 71(4), pp. 344–349. doi: 10.3109/00365513.2011.568630.

Sekhri, T., Juhi, J., Wilfred, R., Kanwar, R., Sethi, J., Bhadra, K., Nair, S. and Singh, S. (2016) 'Trimester specific reference intervals for thyroid function tests in normal Indian pregnant women', *Indian Journal of Endocrinology and Metabolism*. Medknow Publications, 20(1), pp. 101–107. doi: 10.4103/2230-8210.172239.

Singh, P. K., Parvin, C. A. and Gronowski, A. M. (2003) 'Establishment of reference intervals for markers of fetal thyroid status in amniotic fluid', *Journal of Clinical Endocrinology and Metabolism*, 88(9), pp. 4175–4179. doi: 10.1210/jc.2003-030522.

Soldin, O. P., Soldin, D. and Sastoque, M. (2007) 'Gestation-Specific Thyroxine and Thyroid Stimulating Hormone Levels in the United States and Worldwide', *The Drug Monitor*, 29(5), pp. 553–559. doi: 10.1016/j.cortex.2009.08.003.Predictive.

Soldin, O. P., Tractenberg, R. E., Hollowell, J. G., Jonklaas, J., Janicic, N. and Soldin, S. J. (2004) 'Trimester-specific changes in maternal thyroid hormone, thyrotropin, and thyroglobulin concentrations during gestation: Trends and associations across trimesters in iodine sufficiency', *Thyroid*, 14(12), pp. 1084–1090. doi: 10.1089/thy.2004.14.1084.

Supplementary Table 1: Female TSH reference intervals (2.5th and 97.5th percentiles) published from 2000 according to reproductive life stage

Soldin, S. J., Cheng, L. L., Lam, L. Y., Werner, A., Le, A. D. and Soldin, O. P. (2010) ‘Comparison of FT4 with log TSH on the Abbott Architect ci8200: Pediatric reference intervals for free thyroxine and thyroid- stimulating hormone’, *Clin Chim Acta*, 411(0), pp. 250–252. doi: 10.1038/jid.2014.371.

Springer, D., Bartos, V. and Zima, T. (2014) ‘Reference intervals for thyroid markers in early pregnancy determined by 7 different analytical systems’, *Scandinavian Journal of Clinical and Laboratory Investigation*, 74(2), pp. 95–101. doi: 10.3109/00365513.2013.860617.

Springer, D., Zima, T. and Limanova, Z. (2009) ‘Reference intervals in evaluation of maternal thyroid function during the first trimester of pregnancy’, *European Journal of Endocrinology*, 160(5), pp. 791–797. doi: 10.1530/EJE-08-0890.

Sriphrapradang, C., Pavarangkoon, S., Jongjaroenprasert, W., Chailurkit, L. O., Ongphiphadhanakul, B. and Aekplakorn, W. (2014) ‘Reference ranges of serum TSH, FT4 and thyroid autoantibodies in the Thai population: The national health examination survey’, *Clinical Endocrinology*, 80(5), pp. 751–756. doi: 10.1111/cen.12371.

Stricker, Reto, Echenard, M., Eberhart, R., Chevailler, M. C., Perez, V., Quinn, F. A. and Stricker, Rn (2007) ‘Evaluation of maternal thyroid function during pregnancy: The importance of using gestational age-specific reference intervals’, *European Journal of Endocrinology*, 157(4), pp. 509–514. doi: 10.1530/EJE-07-0249.

Su, P. Y., Huang, K., Hao, J. H., Xu, Y. Q., Yan, S. Q., Li, T., Xu, Y. H. and Tao, F. B. (2011) ‘Maternal thyroid function in the first twenty weeks of pregnancy and subsequent fetal and infant development: A prospective population- based cohort study in China’, *Journal of Clinical Endocrinology and Metabolism*, 96(10), pp. 3234–3241. doi: 10.1210/jc.2011-0274.

Su, Q., Zhang, S., Hu, M., Wang, Q., Liu, N., Shen, H., Zhang, Y. and Zhang, M. (2019) ‘Reference Range and Sociodemographic Characteristics of TSH among Reproductive Age Women in Rural China’, *Biological Trace Element Research*. Biological Trace Element Research, 189(2), pp. 336–343. doi: 10.1007/s12011-018-1480-1.

Takeda, K., Mishiba, M., Sugiura, H., Nakajima, A., Kohama, M. and Hiramatsu, S. (2009) ‘Evaluated reference intervals for serum free thyroxine and thyrotropin using the conventional outliner rejection test without regard to presence of thyroid antibodies and prevalence of thyroid dysfunction in japanese subjects’, *Endocrine Journal*, 56(9), pp. 1059–1066. doi: 10.1507/endocrj.K09E-123.

Tozzoli, R., D’Aurizio, F., Metus, P., Steffan, A., Mazzon, C. and Bagnasco, M. (2018) ‘Reference intervals for thyrotropin in an area of Northern Italy: the Pordenone thyroid study (TRIPP)’, *Journal of Endocrinological Investigation*. Springer International Publishing, 41(8), pp. 985–994. doi: 10.1007/s40618-018-0825-0.

Valdés, S., Maldonado-Araque, C., Lago-Sampedro, A., Lillo-Muñoz, J. A., Garcia-Fuentes, E., Perez-Valero, V., Gutiérrez-Repiso, C., Garcia-Escobar, E., Goday, A., Urrutia, I., Peláez, L., Calle-Pascual, A., Bordiú, E., Castaño, L., Castell, C., Delgado, E., Menéndez, E., Franch-Nadal, J., Gaztambide, S., *et al*. (2017) ‘Reference values for TSH may be inadequate to define hypothyroidism in persons with morbid obesity: Di@bet.es study’, *Obesity*, 25(4), pp. 788–793. doi: 10.1002/oby.21796.

Velayutham, K., Selvan, S. S. A. and Unnikrishnan, A. G. (2015) ‘Prevalence of thyroid dysfunction among young females in a South Indian population.’, *Indian journal of endocrinology and metabolism*. Wolters Kluwer -- Medknow Publications, 19(6), pp. 781–4. doi: 10.4103/2230-8210.167546.

Veltri, F., Belhomme, J., Kleynen, P., Grabczan, L., Rozenberg, S., Pepersack, T. and Poppe, K. (2017) ‘Maternal thyroid parameters in pregnant women with different ethnic backgrounds: Do ethnicity-specific reference ranges improve the diagnosis of subclinical hypothyroidism?’, *Clinical Endocrinology*, 86(6), pp. 830–836. doi: 10.1111/cen.13340.

Völzke, H., Alte, D., Kohlmann, T., Lüdemann, J., Nauck, M., John, U. and Meng, W. (2005) ‘Reference intervals of serum thyroid function tests in a previously iodine-deficient area’, *Thyroid*, 15(3), pp. 279–285. doi: 10.1089/thy.2005.15.279.

Wang, D., Li, D., Guo, X., Yu, S., Qiu, L., Cheng, X., Xu, T., Li, H. and Liu, H. (2018) ‘Effects of sex, age, sampling time, and season on thyroid-stimulating hormone concentrations: A retrospective study’, *Biochemical and Biophysical Research Communications*. Elsevier Ltd, 506(3), pp. 450–454. doi: 10.1016/j.bbrc.2018.10.099.

Supplementary Table 1: Female TSH reference intervals (2.5th and 97.5th percentiles) published from 2000 according to reproductive life stage

Wang, Y., Wu, X., Hong, K., Fu, X., Chen, T., Zhu, M. and Ye, H. (2019) 'Establishment of reference intervals for thyroid hormones in premature infants beyond the first week of life using Beckman Coulter Unicel DxI 800', *Clinical Biochemistry*. Elsevier, 74(August), pp. 19–23. doi: 10.1016/j.clinbiochem.2019.09.003.

Wang, Y., Zhang, Y. X., Zhou, Y. L. and Xia, J. (2017) 'Establishment of reference intervals for serum thyroid-stimulating hormone, free and total thyroxine, and free and total triiodothyronine for the Beckman Coulter DxI-800 analyzers by indirect method using data obtained from Chinese population in Zhejiang', *Journal of Clinical Laboratory Analysis*, 31(4), pp. 1–6. doi: 10.1002/jcla.22069.

Wang, Yanling, Zhang, Z., Ge, P., Wang, Yibo and Wang, S. (2009) 'Iodine status and thyroid function of pregnant, lactating women and infants (0-1 yr) residing in areas with an effective Universal Salt Iodization program.', *Asia Pacific journal of clinical nutrition*, 18(1), pp. 34–40. doi: 10.6133/apjcn.2009.18.1.06.

Waring, A. C., Arnold, A. M., Newman, A. B., Buzková, P., Hirsch, C. and Cappola, A. R. (2012) 'Longitudinal changes in thyroid function in the oldest old and survival: The cardiovascular health study all-stars study', *Journal of Clinical Endocrinology and Metabolism*, 97(11), pp. 3944–3950. doi: 10.1210/jc.2012-2481.

Xing, J., Yuan, E., Li, J., Zhang, Y., Meng, X., Zhang, X., Rong, S., Lv, Z., Tian, Y. and Jia, L. (2016) 'Trimester- and assay-specific thyroid reference intervals for pregnant women in China', *International Journal of Endocrinology*, 2016(January 2013). doi: 10.1155/2016/3754213.

Yan, Y. Q., Dong, Z. L., Dong, L., Wang, F. R., Yang, X. M., Jin, X. Y., Lin, L. X., Sun, Y. N. and Chen, Z. P. (2011) 'Trimester-and method-specific reference intervals for thyroid tests in pregnant Chinese women: Methodology, euthyroid definition and iodine status can influence the setting of reference intervals', *Clinical Endocrinology*, 74(2), pp. 262–269. doi: 10.1111/j.1365-2265.2010.03910.x.

Yang, X., Meng, Y., Zhang, Y., Zhang, C., Guo, F., Yang, S., Ding, R., Fan, J. X. and Lyu, P. (2019) 'Thyroid function reference ranges during pregnancy in a large Chinese population and comparison with current guidelines', *Chinese Medical Journal*, 132(5), pp. 505–511. doi: 10.1097/CM9.0000000000000051.

Yeo, C. P., Khoo, D. H. C., Eng, P. H. K., Tan, H. K., Yo, S. L. and Jacob, E. (2001) 'Prevalence of gestational thyrotoxicosis in Asian women evaluated in the 8th to 14th weeks of pregnancy: Correlations with total and free beta human chorionic gonadotrophin', *Clinical Endocrinology*, 55(3), pp. 391–398. doi: 10.1046/j.1365-2265.2001.01353.x.

Yoshihara, A., Noh, J. Y., Ohye, H., Sato, S., Sekiya, K., Kosuga, Y., Suzuki, M., Matsumoto, M., Kunii, Y., Watanabe, N., Mukasa, K., Ito, Kunihiko and Ito, Koichi (2011) 'Reference limits for serum thyrotropin in a Japanese population', *Endocrine Journal*, 58(7), pp. 585–588. doi: 10.1507/endocrj.K11E-082.

Zarghami, N., Rohbani-Noubar, M. and Khosrowbeygi, A. (2005) 'Thyroid hormones status during pregnancy in normal Iranian women', *Indian Journal of Clinical Biochemistry*, 20(2), pp. 182–185. doi: 10.1007/BF02867424.

Zhang, D., Cai, K., Wang, G., Xu, S., Mao, X., Zheng, A., Liu, C. and Fan, K. (2019) 'Trimester-specific reference ranges for thyroid hormones in pregnant women', *Medicine*, 98(4), p. e14245. doi: 10.1097/MD.0000000000014245.

Zhang, X., Yao, B., Li, Chenyan, Mao, J., Wang, W., Xie, X., Teng, X., Han, C., Zhou, W., Li, Chenyang, Xu, B., Bi, L., Meng, T., Du, J., Zhang, S., Gao, Z., Yang, L., Fan, C., Teng, W., *et al*. (2016) 'Reference Intervals of Thyroid Function during Pregnancy: Self-Sequential Longitudinal Study Versus Cross-Sectional Study', *Thyroid*, 26(12), pp. 1786–1793. doi: 10.1089/thy.2016.0002.
